# Dissection of mRNA ac^4^C acetylation modifications in AC and *Nr* fruits: insights into the regulation of fruit ripening by ethylene

**DOI:** 10.1186/s43897-024-00082-7

**Published:** 2024-02-19

**Authors:** Lili Ma, Yanyan Zheng, Zhongjing Zhou, Zhiping Deng, Jinjuan Tan, Chunmei Bai, Anzhen Fu, Qing Wang, Jinhua Zuo

**Affiliations:** 1 Institute of Agri-food Processing and Nutrition, Beijing Academy of Agriculture and Forestry Sciences, Beijing Key Laboratory of Fruits and Vegetable Storage and Processing, Key Laboratory of Vegetable Postharvest Processing of Ministry of Agriculture and Rural Areas, State Key Laboratory of Vegetable Biobreeding, Beijing Vegetable Research Center, Beijing Academy of Agriculture and Forestry Science, Beijing, 100097 China; 2 Zhejiang Academy of Agricultural Sciences, Hangzhou, 310021 China; 3 School of Food and Health, Beijing Technology and Business University (BTBU), Beijing, 100048 China

**Keywords:** ac^4^C modification, Tomato, Ethylene, Fruit ripening, mRNA

## Abstract

N^4^-acetylcytidine (ac^4^C) modification of mRNA has been shown to be present in plant RNAs, but its regulatory function in plant remains largely unexplored. In this study, we investigated the differentially expressed mRNAs, lncRNAs and acetylation modifications of mRNAs in tomato fruits from both genotypes. By comparing wild-type (AC) tomato and the ethylene receptor-mutant (*Nr*) tomato from mature green (MG) to six days after the breaker (Br6) stage, we identified differences in numerous key genes related to fruit ripening and observed the corresponding lncRNAs positively regulated the target genes expression. At the post-transcriptional level, the acetylation level decreased and increased in AC and *Nr* tomatoes from MG to Br6 stage, respectively. The integrated analysis of RNA-seq and ac^4^C-seq data revealed the potential positive role of acetylation modification in regulating gene expression. Furthermore, we found differential acetylation modifications of certain transcripts (*ACO*, *ETR*, *ERF*, *PG*, *CesA*, *β-Gal*, *GAD*, *AMY*, and *SUS*) in AC and *Nr* fruits which may explain the differences in ethylene production, fruit texture, and flavor during their ripening processes. The present study provides new insights into the molecular mechanisms by which acetylation modification differentially regulates the ripening process of wild-type and mutant tomato fruits deficient in ethylene signaling.

**Graphical Abstract:**

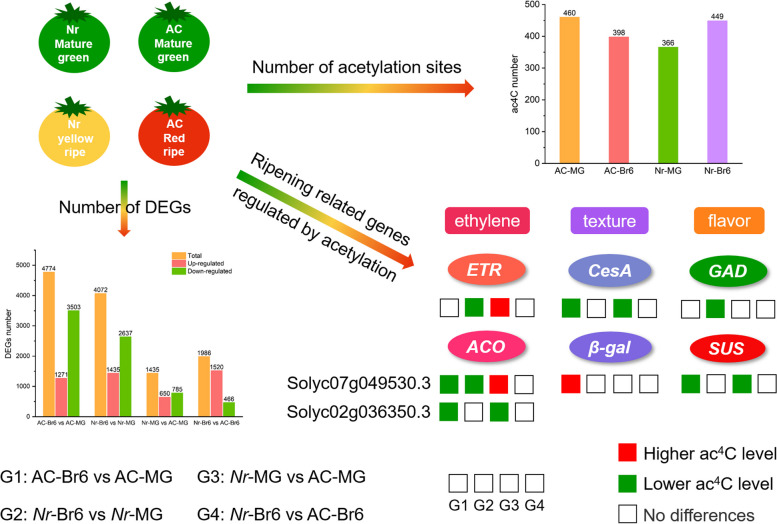

**Supplementary Information:**

The online version contains supplementary material available at 10.1186/s43897-024-00082-7.

## Core

The expression of most DEGs decreased with tomato ripening. Notably, the expression of DEGs in *Nr* fruits was higher than those in AC fruits at Br6 stage. Correspondingly, the overall mRNA acetylation level in *Nr* fruit was higher than that of AC fruit at Br6 stage, indicating that the inhibited ripening phenotype of *Nr* fruit may be related to increase in acetylation and mRNAs expression. Therefore, acetylation may be ripening related and play a regulatory role in tomato fruit ripening.

## Gene & accession numbers

Sequence data from this article can be found in the database of the National Center for Biotechnology Information (NCBI) under the accession numbers: PRJNA1053495 and PRJNA1053422.

## Introduction

Tomato (*Solanum lycopersicum* L.) is a popular fleshy fruit, known for its vibrant color, attractive flavor, and nutritional value, providing essential sugars, organic acids, dietary fibers, and other health-supporting nutrients (Wang et al. 2022; Zhang et al. 2023). The tomato ripening process involves major physiological and biochemical changes, such as pigments accumulation, chlorophyll degradation leading to color change, cell wall depolymerization causing fruit softening, the accumulation of sugars and acids contributing to unique flavor, and the biosynthesis of characteristic nutrients (Giovannoni et al. 2017; Wang and Seymour 2022; Zhu et al. 2022). These processes are tightly regulated through the modulation of expression of many genes (Bres et al. 2022; Wang et al. 2022). The molecular mechanism of ripening depends on the coordination of transcription factors (Gapper et al. 2013; Chen et al. 2020), plant hormones (Kumar et al. 2014; Fenn and Giovannoni 2021; Fu et al. 2022), DNA methylation (Tang et al. 2020; Yao et al. 2022), and histone modifications (Lü et al. 2018). Together, these factors constitute the comprehensive regulatory network governing fruit ripening (Giovannoni et al. 2017; Brumos 2021, Li et al. 2021).

Recent advances in high-throughput technology have led to the exploration of the epitranscriptome, revealing posttranscriptional controls involved in RNA regulation as critical players in gene expression regulation, complementing the transcriptional regulation (Frye et al. 2016; Zhao et al. 2017; Shi et al. 2020; Wang et al. 2022). Although post-transcriptional base modifications in cell mRNAs mostly retain the underlying genetic code (Dominissini and Rechavi 2018), they bear immense biological consequences. The continuous research on mRNA base modifications (Zheng et al. 2013; Vissers et al. 2020) has rekindled recognition of the universality and biological significance of internal mRNA modifications, influencing gene expression and potentially playing vital roles in the molecular regulation, including fruit ripening (Choi and Meyer 2018).

Among the unexplored mRNA base modifications, N^4^-acetylcytidine (ac^4^C) stands out as a highly conserved RNA acetylation modification present in all domains of life (Sharma et al. 2015; Boccaletto et al. 2022). While extensively present within tRNA and rRNA, ac^4^C has also been identified and studied in eukaryotic mRNA (Ito et al. 2014a, b). The ac^4^C events are catalyzed by the N-acetyltransferase 10 (NAT10) enzyme or its homologs, which possess both RNA binding and acetyltransferase activities (Chimnaronk et al. 2009; Ito et al. 2014b). Early studies detected low levels of ac4C in eukaryotic mRNA (Dong et al. 2016), and subsequently, comprehensive mapping confirmed the existence of ac^4^C in mRNA. This discovery expanded the repertoire of mRNA base modifications and highlighted the role of ac^4^C in mRNA translation regulation. Specifically, ac^4^C was identified as a widespread mark in cellular mRNAs, regulating mRNA stability and promoting translation efficiency by facilitating tRNA decoding (Arango et al. 2018). Importantly, the function of ac^4^C in mRNA depends on its location, with acetylation within the coding sequence (CDS) regions promoting transcript stability. Furthermore, acetylation of ac^4^C within mRNA was found to affect mRNA stability and translation directly, independent of the effects caused by acetylation in tRNA and rRNA (Arango et al. 2018; Li et al. 2019). Additionally, ac4C acetylation modifications have been confirmed in Arabidopsis and rice mRNA, suggesting a possible conservation of RNA acetylation events in plants (Li et al. 2023).

Given the critical role of epigenetics in regulating tomato fruit ripening and the growing understanding of mRNA modifications' influence on gene expression (Bucher et al. 2018; Choi and Meyer 2018), the contribution of ac^4^C acetylation modifications in mRNA to fruit ripening regulation requires exploration. In this study, we investigated the effects of ac^4^C acetylation modifications on gene expression during tomato fruit ripening by analyzing two types of tomatoes (AC and the classical mutant *Nr*) at different maturity stages. This comprehensive and systematic research will enhance our understanding of mRNA modifications and may reveal potential biomarkers and their regulatory functions related to tomato ripening at the epigenetic level.

## Results

### The transcriptome in AC and *Nr* tomato fruits

**Fig. 1:**
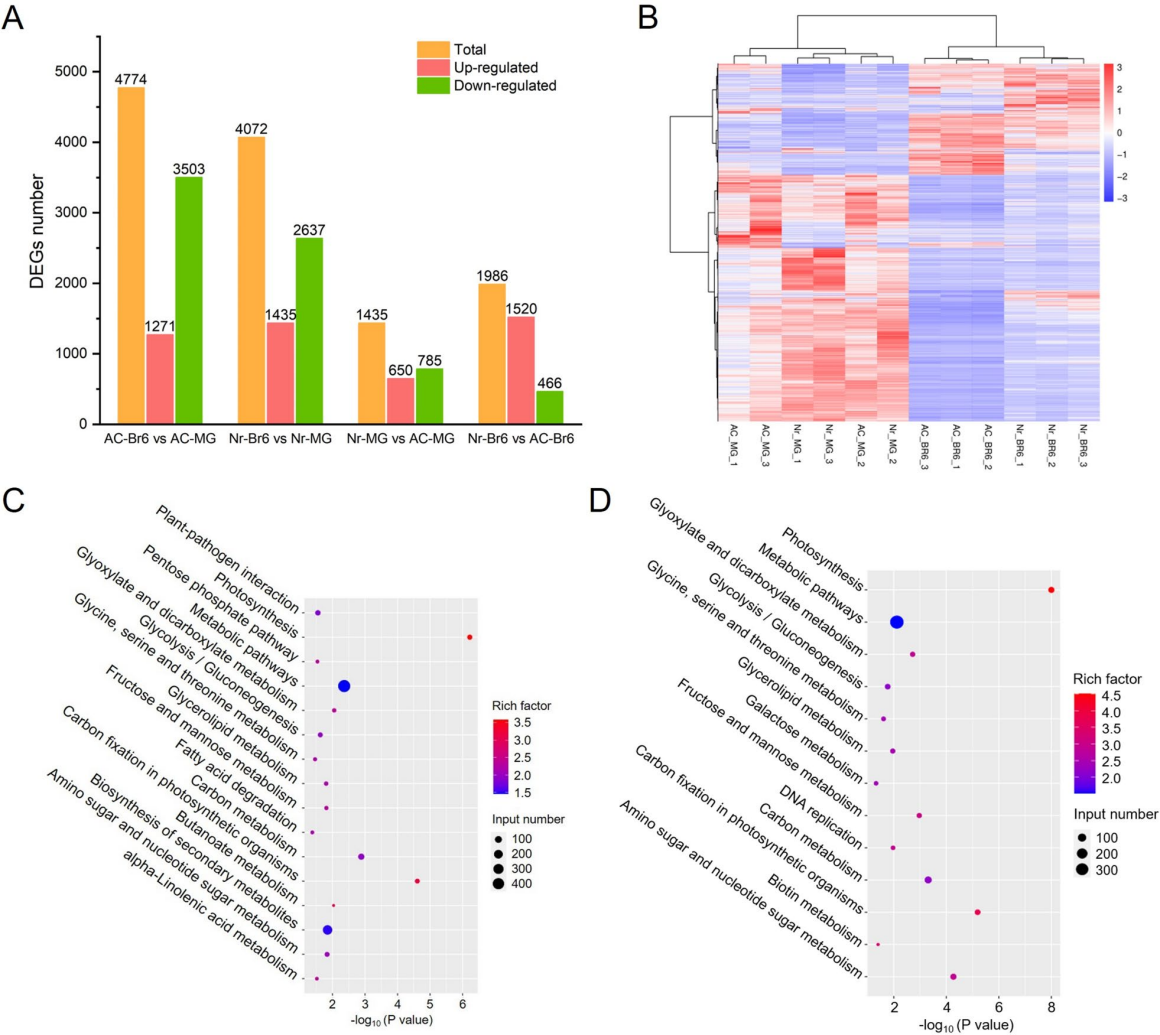
Comparative analysis of differentially expressed genes (DEGs) during the ripening in AC and *Nr* tomato fruits. **A** The number of up-regulated and down-regulated DEGs in AC-Br6 vs AC-MG, *Nr*-Br6 vs *Nr*-MG, *Nr*-MG vs AC-MG and *Nr*-Br6 vs AC-Br6. **B** Heatmap expression profiles of DEGs in four tomato samples (AC-Br6, AC-MG, *Nr*-Br6, *Nr*-MG). **C** The KEGG pathway enrichment of downregulated DEGs in AC-Br6 vs AC-MG. **D** The KEGG pathway enrichment of downregulated DEGs in *Nr*-Br6 vs *Nr*-MG

Transcriptomic analysis revealed that a total of 4774 and 4072 differentially expressed genes (DEGs), including 1271 and 1435 upregulated genes, and 3503 and 2637 downregulated genes, were identified in AC-Br6 vs AC-MG and *Nr*-Br6 vs *Nr*-MG, respectively (Fig. 1A, Table S1), and their expression patterns were shown in the Fig. 1B. KEGG pathway analysis indicated that shared enrichment pathways for the most downregulated DEGs in tomato fruits from both genotypes mainly included photosynthesis, carbon fixation in photosynthetic organisms (Fig. 1C, D). In AC fruit, these downregulated DEGs were mainly enriched in butanoate metabolism pathway, whereas in *Nr* tomato, they enriched in amino sugar, and nucleotide sugar metabolism, and carbon metabolism pathways (Fig. 1C, 1D). For the most upregulated DEGs, the shared enrichment pathways were involved in carotenoid biosynthesis and linoleic acid metabolism (Figure S1). In addition, these upregulated DEGs were mainly enriched in biosynthesis of unsaturated fatty acids and peroxisome in AC tomao, in biosynthesis of secondary metabolites and phenylalanine metabolism in *Nr* tomato (Figure S1).

A total of 1435 and 1986 DEGs were detected in *Nr*-MG vs AC-MG and *Nr*-Br6 vs AC-Br6, respectively, with 785 and 466 DEGs down-regulated, and 650 and 1520 DEGs up-regulated (Fig. 1A, Table S1). In the comparison group *Nr*-MG vs AC-MG, the KEGG pathway analysis indicated that the DEGs were mainly enriched in pathways such as photosynthesis-antenna proteins, starch and sucrose metabolism, metabolic pathways, MAPK signaling, cutin, suberine, and wax biosynthesis, carotenoid biosynthesis, peroxisome (Figure S2). And in the comparison group *Nr*-Br6 vs AC-Br6, the DEGs were primarily enriched in carbon fixation in photosynthetic organisms, photosynthesis-antenna proteins, carbon metabolism, DNA replication, carotenoid biosynthesis, MAPK signaling pathway, and glutathione metabolism pathways (Figure S3).

### The differentially expressed lncRNAs in AC and *Nr* tomato fruits

**Fig. 2:**
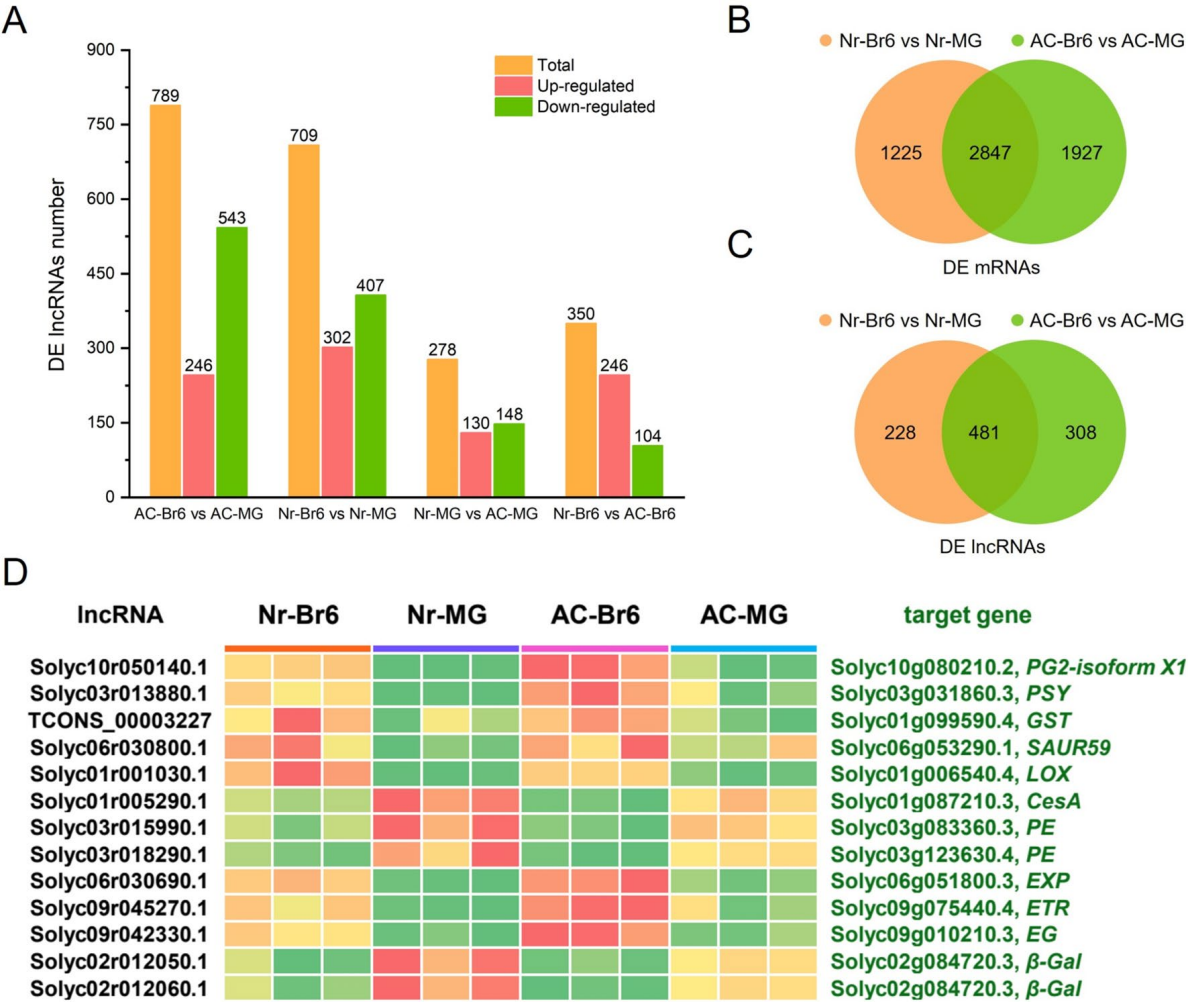
Comparative analysis of DEGs and DE lncRNAs during the ripening in AC and *Nr* tomato fruits. **A** The number of up-regulated and down-regulated DE lncRNAs in AC-Br6 vs AC-MG, *Nr*-Br6 vs *Nr*-MG, *Nr*-MG vs AC-MG and *Nr*-Br6 vs AC-Br6. **B** The number of common and unique DE mRNAs between the *Nr*-Br6 vs *Nr*-MG and AC-Br6 vs AC-MG. **C** The number of common and unique DE lncRNAs between the *Nr*-Br6 vs *Nr*-MG and AC-Br6 vs AC-MG. **D** Cluster heatmaps of common DE lncRNAs targeting common DEGs related to fruit ripening in the two comparison groups of AC-Br6 vs AC-MG and *Nr*-Br6 vs *Nr*-MG

Notably, 789 and 709 differentially expressed (DE) lncRNAs were identified in two comparison groups AC-Br6 vs AC-MG and *Nr*-Br6 vs *Nr*-MG, with 246 and 302 DE lncRNAs showing increased expression, 543 and 407 DE lncRNAs showing decreased expression, respectively (Fig. 2A, Table S2). Importantly, 575 and 504 DEGs were identified as the target genes of 564 and 501 DE lncRNAs in AC and *Nr* tomato fruits, respectively (Table S3). The majority of DE lncRNAs displayed similar expression patterns to their DE target genes (Table S3), which indicated that lncRNAs may positively regulate target gene expression. Additionally, KEGG pathway analysis indicated that the target genes of DE lncRNAs were mainly enriched in carotenoid biosynthesis, porphyrin and chlorophyll metabolism, alpha-linoleic acid metabolism, photosynthesis-antenna proteins, carbon fixation in photosynthetic organisms and photosynthesis pathways in the comparison groups AC-Br6 vs AC-MG (Figure S4). However, the target genes were mainly associated with pathways related to phenylalanine metabolism, glutathione metabolism, carotenoid biosynthesis, photosynthesis-antenna proteins, carbon fixation in photosynthetic organisms, and photosynthesis in the *Nr*-Br6 vs *Nr*-MG (Figure S5).

We further identified and analyzed 278 and 350 DE lncRNAs in the *Nr*-MG vs AC-MG and *Nr*-Br6 vs AC-Br6 comparison groups, of which 130 and 246 lncRNAs exhibited elevated expression, while 148 and 104 lncRNAs showed reduced expression, respectively (Fig. 2A, Table S2). Notably, 173 and 224 DEGs were identified as the target genes of 172 and 210 DE lncRNAs in the *Nr*-MG vs AC-MG and *Nr*-Br6 vs AC-Br6 comparison groups, respectively (Table S3). Interestingly, the expression trends of these DE lncRNAs and their target genes were completely consistent (Table S3). Additionally, KEGG pathway analysis indicated that the target genes of DE lncRNAs were mainly enriched in photosynthesis-antenna proteins and porphyrin and chlorophyll metabolism pathways, amino sugar and nucleotide sugar metabolism, cutin, suberine, and wax biosynthesis, MAPK signaling pathways in the *Nr*-MG vs AC-MG comparison group (Figure S6). However, in the comparison group *Nr*-Br6 vs AC-Br6, the target genes were significantly enriched in pathways related to carotenoid biosynthesis, glutathione metabolism, propanoate metabolism, photosynthesis-antenna proteins, carbon fixation in photosynthetic organisms, phenylalanine metabolism (Figure S7).

### Comparative analysis of transcriptome between AC and *Nr* fruits ripening processes

To analyze the differences between AC fruit and *Nr* mutant fruits ripening processes, we identified unique and common DEGs between AC-Br6 vs AC-MG and *Nr*-Br6 vs *Nr*-MG, and focused on 2847 shared DEGs in two comparison groups (Fig. 2B, Table S4), the variation trend of 2821 common DEGs in two tomato varieties is consistent, and 26 common DEGs show different expression trends (Table S4). The differential expression of these genes may lead to differences in fruit ripening of AC and *Nr* tomatoes.

Moreover, we analyzed DE lncRNAs and their corresponding target genes between the AC-Br6 vs AC-MG and *Nr*-Br6 vs *Nr*-MG comparison groups. A total of 481 shared DE lncRNAs were found in both AC and *Nr* tomato fruits (Fig. 2C, Table S5), of these, 310 and 309 lncRNAs were downregulated, 171 and 172 lncRNAs were upregulated during the ripening process of AC and *Nr* fruits, respectively, only 1 common lncRNAs (TCONS_00002811) showed different expression trends in AC and *Nr* tomato during fruit ripening (Table S5). In addition, we observed that 297 common DE mRNAs targeted by 339 common DE lncRNAs (Table S6).

Then, we focused on common DEGs and common DE lncRNAs related to fruit ripening, including ethylene-related gene *1-aminocyclopropane-1-carboxylate synthase* (*ACS*) and *ethylene receptor* (*ETR*); color-related genes *phytoene synthase* (*PSY*), *zeta-carotene desaturase* (*ZDS*), *beta-carotene hydroxylase* (*BCH*), *lycopene epsilon cyclase* (*LCYE*); texture-related genes *pectate lyase* (*PL*), *pectin acetylesterase* (*PAE*), *pectinesterase* (*PE*), *polygalacturonase* (*PG*), *endoglucanase* (*EG*), *beta-galactosidase* (*β-Gal*), *beta-glucosidase* (*β-Glu*), *expansin* (*EXP*), *cellulose synthase* (*CesA*) and *β-D-xylosidase*; flavor-related genes *glutamate decarboxylase* (*GAD*), *sucrose-phosphate synthase* (*SPS*), *sucrose synthase* (*SUS*), *starch synthase* (*SS*), *alcohol acyl transferase* (*AAT*), *malic enzyme* (*ME*), *phenylalanine ammonia-lyase* (*PAL*), *lipoxygenase* (*LOX*), *malate dehydrogenase* (*MDH*), *cell-wall invertase* (*CWIN*), and *bidirectional sugar transporter SWEET* (Table S7). Notably, the numerous common DE lncRNAs (Solyc09r045270.1, Solyc03r013880.1, Solyc01r005290.1, Solyc03r015990.1 and Solyc03r018290.1, Solyc09r042330.1, Solyc06r030690.1, Solyc02r012050.1 and Solyc02r012060.1, Solyc01r001030.1) targeting *ETR*, *PSY*, *CesA*, *PE*, *EG*, *EXP*, *β-Gal*, *LOX*, respectively, exhibited similar expression patterns and fold change to theirs target gene (Fig. 2D, Table S8).

### The RNA ac^4^C modification during ripening in AC and *Nr* tomato fruits

**Fig. 3:**
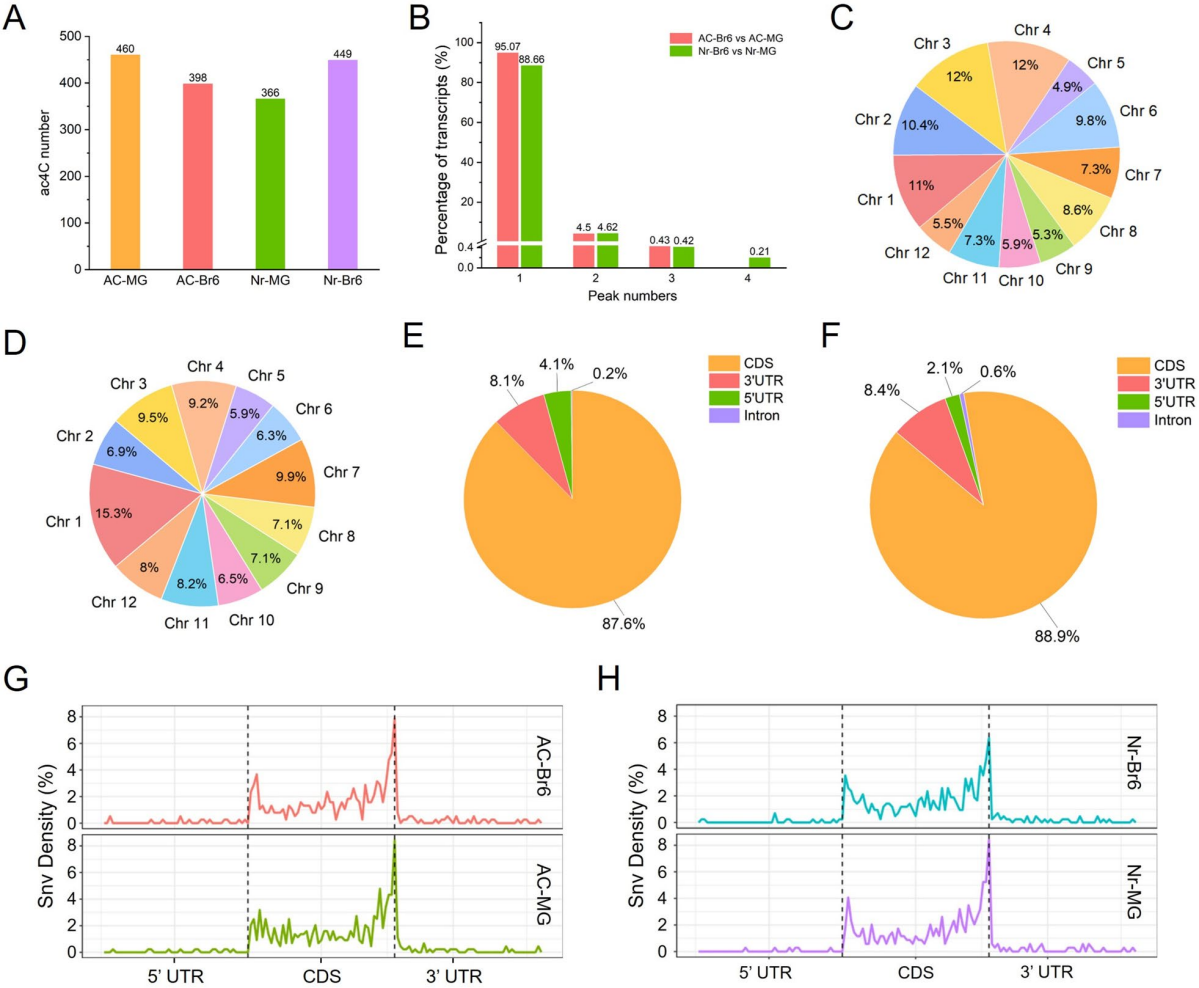
Overview of ac^4^C acetylation profiles in AC and *Nr* tomatoes. **A** Number of ac^4^C sites detected in four tomato samples (AC-MG, AC-Br6, *Nr*-MG, *Nr*-Br6). **B** Proportions of the ac^4^C-modified transcripts containing different ac^4^C peak numbers in AC and *Nr* tomatoes fruit ripening processes. **C** The pie chart presenting the distribution proportion of differential ac^4^C modification within mRNA across the 12 chromosomes in AC-Br6 vs AC-MG. **D** The distribution proportion of differential ac^4^C modification within mRNA across the 12 chromosomes in *Nr*-Br6 vs *Nr*-MG. **E** Pie chart displaying the fraction of differential ac^4^C peak summits in four non-overlapping transcript segments in AC-Br6 vs AC-MG. **F** The fraction of differential ac^4^C peak summits in four non-overlapping transcript segments in *Nr*-Br6 vs *Nr*-MG. **G-H** Metagenomic profiles of peak summit distributions along the transcripts composed of non-overlapping segments (5’UTR, CDS, and 3’UTR). UTR, untranslated region; CDS, coding sequence

We also investigated the changes in ac^4^C levels during AC and *Nr* tomato fruits ripening, and detected 460 acetylation sites in AC-MG fruit samples, while in AC-Br6 samples, the number of acetylation sites decreased to 398, indicating an overall decline in ac^4^C levels as AC fruit ripening progressed (Fig. 3A). Conversely, we found that the overall ac^4^C levels increased as *Nr* tomato fruit ripening, with 366 and 449 acetylation sites in *Nr*-MG and *Nr*-Br6 fruit samples, respectively (Fig. 3A).

In order to deeply understand the different ac^4^C acetylation modification events during ripening in AC and *Nr* tomato fruits, we identified and analyzed differential ac^4^C sites (DAS) during fruit ripening in two tomatoes. A total of 521 and 506 DAS were detected in AC-Br6 vs AC-MG and *Nr*-Br6 vs *Nr*-MG, respectively (Table S9). Specifically, during the ripening process of AC fruits, 492 DAS were found on 467 transcripts, with the acetylation modification level of 220 sites on 215 transcripts being upregulated, and the acetylation modification level of 272 sites on 264 transcripts being downregulated (Table S10). Notably, the DAS with low acetylation levels were more abundant than sites with high acetylation levels, indicating a global reduction trend in RNA (ac^4^C) acetylation during fruit ripening in AC tomato. While during the ripening process of *Nr* fruits, 476 DAS located on 447 transcripts, of these, the acetylation modification level of 268 sites on 257 transcripts was upregulated, and the acetylation modification level of 208 sites on 203 transcripts was downregulated (Table S10). Unlike AC fruit, the ac^4^C sites with high acetylation levels were more abundant than sites with low acetylation levels, indicating a global increased trend in RNA (ac^4^C) acetylation during fruit ripening in *Nr* tomato fruit.

In AC fruit, most transcripts (444, 95.07%) containing ac^4^C modification had one ac^4^C peak, while 21 (4.50%) exhibited two ac^4^C peaks, and only 2 (0.43%) exhibited three peaks (Fig. 3B, Table S10). While in *Nr* fruit, the majority of transcripts (88.66%) containing ac^4^C modification displayed one ac^4^C peak, while 4.62% had two ac^4^C peaks, 0.42% exhibited three ac^4^C peaks, and 0.21% showed four peaks which is not found in AC fruit (Fig. 3B, Table S10), indicating that certain transcripts in *Nr* fruit have more acetylation modification sites compared to AC fruit.

At the genomic level, the DAS in AC fruit were not uniformly distributed across all chromosomes, with chromosomes 3 and 4 having the largest number of acetylated sites, while chromosome 5 had the fewest acetylated sites (Fig. 3C, Table S10). However, for the *Nr* tomato fruit, our results revealed a broad distribution of differential ac^4^C modification across all chromosomes of the genome, with chromosome 1 having the most acetylated sites, approximately twice as many as other chromosomes (Fig. 3D, Table S10).

The mRNAs were divided into four non-overlapping transcript segments to confirm the distribution of ac^4^C. In the comparison group of AC-Br6 vs AC-MG, we found that most of the DAS (431, 87.6%) were mainly located within the CDS, while a few ac^4^C sites were located within the 3' untranslated region (3’UTR) (40, 8.1%) and 5'UTR (20, 4%), only one ac^4^C site (0.2%) was located in the intron region (Fig. 3E, Table S10). Similarly, in *Nr* tomato fruits at the Br6 vs MG stages, the majority of the DAS (423, 88.9%) were mainly located within the CDS, while some ac^4^C sites were found in the 3'UTR (40, 8.4%) and 5'UTR (10, 2.1%), with only three ac^4^C sites (0.6%) located in the intron region (Fig. 3F, Table S10). The metagenomic profiles of ac^4^C peaks in AC and *Nr* tomatoes indicated a high enrichment of ac^4^C modifications within the CDS and around the stop codon (Fig. 3G, H).

**Fig. 4:**
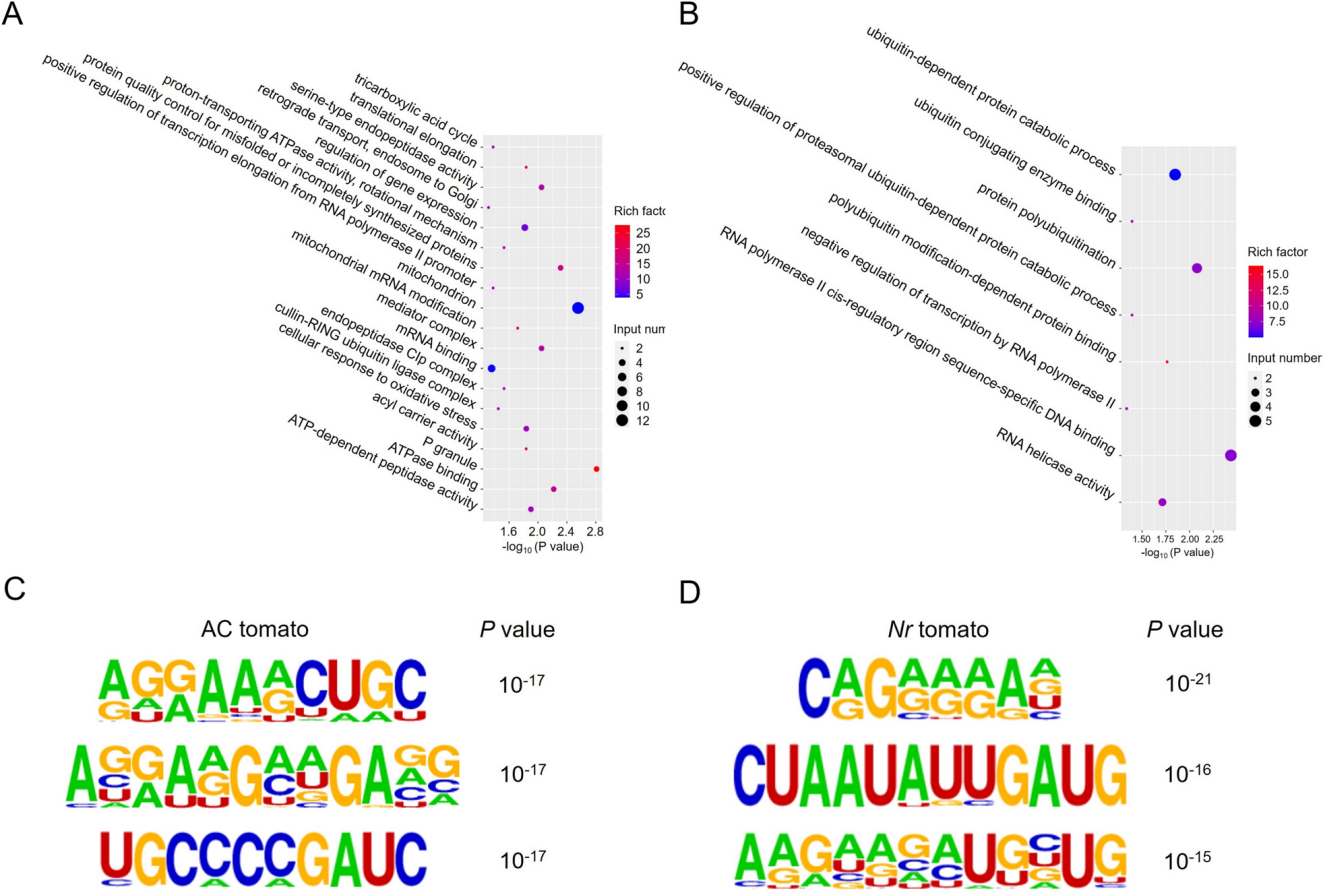
Gene Ontology (GO) analysis for the ac^4^C-containing transcripts and identification of sequence motifs. **A** GO analysis for the ac^4^C-containing transcripts identified in AC-Br6 vs AC-MG comparison group. **B** GO analysis for the ac^4^C-containing transcripts identified in *Nr*-Br6 vs *Nr*-MG comparison group. **C** Sequence motifs identified within ac^4^C peaks by HOMER in AC tomato fruit. **D** Sequence motifs identified within ac^4^C peaks in *Nr* tomato fruit

Moreover, we performed GO enrichment analysis of ac^4^C-containing transcripts. In AC fruit, the differential ac^4^C site-related genes were mainly enriched in biological processes (BP) related to protein quality control for misfolded or incompletely synthesized proteins, cellular response to oxidative stress, translational elongation, and regulation of gene expression (Fig. 4A). The most relevant GO terms associated with molecular function (MF) were ATPase binding, serine-type endopeptidase activity, ATP-dependent peptidase activity, and acyl carrier activity (Fig. 4A). Whereas in *Nr* fruit, GO terms analysis showed that differential ac^4^C site-related genes involved in BP and MF were mainly enriched in RNA polymerase II cis-regulatory region, sequence-specific DNA binding, protein polyubiquitination, ubiquitin-dependent protein catabolic process, polyubiquitin modification-dependent protein binding, RNA helicase activity, etc (Fig. 4B). The results above indicated different potential functions of ac^4^C modification during the ripening of two tomato fruits.

Furthermore, we performed clustering of ac^4^C peaks using HOMER to identify consensus motifs in ac^4^C peak sites and identified several sequence motifs significantly enriched within the ac^4^C peaks, the top three motifs were AGRAARCTGC (R represents A or G), AGGARGMWGAVG (M represents A or C; W represents A or T; V represents G, A or C) and TGCCCCGATC in AC tomato fruit (Fig. 4C). In addition, CAGRRRAD, CTAATATTGATG and AAGAAGATGBTG were the top three motifs identified in *Nr* tomato fruit (Fig. 4D), these motifs are similar to motifs found in many classes of transcription factors (TFs).

### The integrated analysis of RNA-seq and ac^4^C-seq data during ripening in AC and *Nr* tomato fruits

**Fig. 5:**
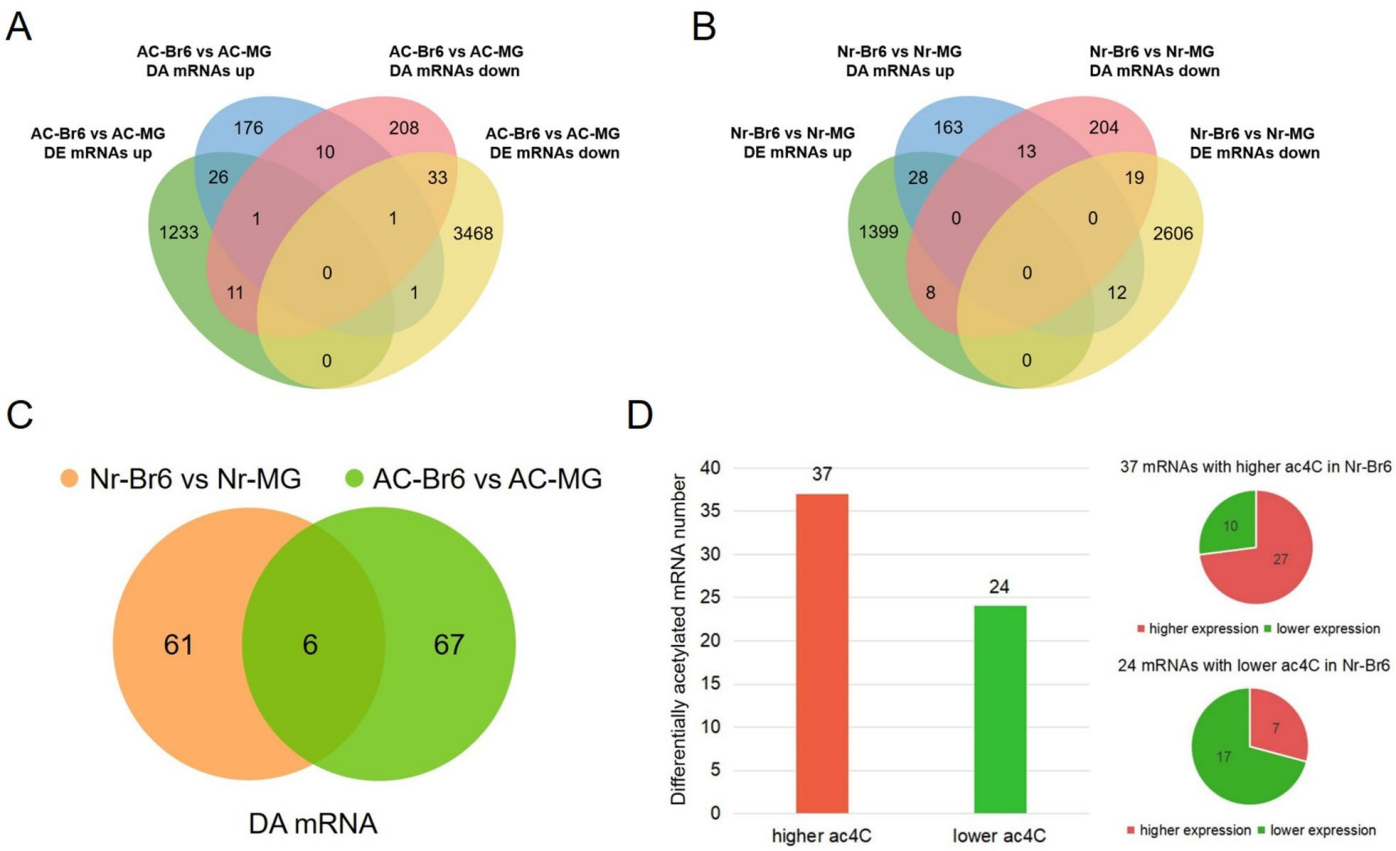
The integrated analysis of DA mRNA and DE mRNA during ripening in AC and *Nr* tomato fruits. **A** Correlation analysis of gene expression and mRNA acetylation (ac^4^C) in AC-Br6 vs AC-Br6. **B** Correlation analysis of gene expression and mRNA acetylation (ac^4^C) in *Nr*-Br6 vs *Nr*-MG. **C** Venn diagram showing the shared and specific DA mRNAs in the comparison groups of AC-Br6 vs AC-MG and *Nr*-Br6 vs *Nr*-MG.** D** The bar chart showing the number of unique differentially expressed mRNAs in *Nr*-Br6 vs *Nr*-MG. The two pie chart displaying the number of mRNAs with higher and lower ac^4^C levels in mRNAs with higher and lower expression in *Nr*-Br6, respectively

We conducted a combined analysis of differentially acetylated mRNAs (DA mRNAs) and differentially expressed mRNAs (DE mRNAs) in two tomatoes. During ripening in AC tomato fruit, we observed 73 mRNAs that showed differences in both ac^4^C acetylation modification and expression level (Fig. 5A, Table S11). Among the 46 mRNAs with lower ac^4^C levels, 34 also showed lower expression levels (Fig. 5A, Table S11). Among the 29 mRNAs with higher ac^4^C levels, 27 showed higher expression levels (Fig. 5A, Table S11). Most notably, in AC fruit, the changes in the expression levels of several DEGs *ACO*, *SUS*, *CesA* and *β-Gal* related to fruit ripening were consistent with the changes in the acetylation modification levels on corresponding transcripts (Table S11).

On the other hand, a total of 67 mRNAs exhibited differences in both ac^4^C acetylation modification level and expression levels during the ripening in *Nr* tomato fruit (Fig. 5B, Table S11). Among the 40 mRNAs with higher ac^4^C levels, 28 mRNAs showed a higher expression level. And among the 27 mRNAs with lower ac^4^C levels, 19 mRNAs showed a lower expression level (Fig. 5B, Table S11). Interesting, we found that the changes in the expression levels of several DEGs *ERF*, *AMY*, *CesA*, *PG*, *GAD* were consistent with the changes in the acetylation modification levels on corresponding transcripts, except *ACO* and *ETR* (Table S11). These results support the conclusion that ac^4^C acetylation on mRNA may play a significant role in promoting target gene expression (Arango et al. 2018), thereby regulating tomato fruit ripening.

### Comparative analysis of differential acetylation modification within DE mRNAs between AC and *Nr* fruits ripening processes

The comparative analysis of acetylation modification revealed that among the overlap of DA mRNAs and DE mRNAs, only 6 mRNAs were common in two comparison groups *Nr*-Br6 vs *Nr*-MG and AC-Br6 vs AC-MG, and a total of 67 and 61 DA mRNAs expressed only in AC and *Nr* tomato fruit, respectively (Fig. 5C, Table S12). Furthermore, we focused on the overlap of DA mRNAs and DE mRNAs that were specifically present in *Nr* fruit. Among the specific DE mRNAs in the *Nr* mutant, 34 mRNAs showed increased expression, and 27 of these mRNAs also exhibited an upward trend in acetylation levels (Fig. 5D, Table S12). Besides, 27 mRNAs displayed decreased expression, and 17 of them showed a corresponding downward trend in acetylation levels (Fig. 5D, Table S12). The results suggested a positive correlation between mRNA acetylation levels and expression levels. Notably, some genes related to fruit ripening, such as *ACO* (Solyc07g049530.3), *ETR*, *ERF*, *ARF*, *PG*, *GAD*, *CesA*, and *AMY*, were only found in *Nr* tomato, other four genes including *ACO* (Solyc02g036350.3), *CesA*, *β-gal* and *SUS* were only found in AC tomato (Table S12). The result showed that the expression of these specific genes may be affected by ac^4^C acetylation modifications within their corresponding mRNAs in tomato fruit during ripening. However, further exploration is needed to understand how ac^4^C acetylation modification regulates gene expression.

### The RNA ac^4^C modification at the MG and Br6 stage in AC and *Nr* tomato fruits

We also explored the ac^4^C acetylation modification events of tomato fruits from both genotypes at the MG and Br6 stages, and observed the number of acetylation sites in *Nr* fruits was lower than that in AC fruits at the MG stage, while the situation was exactly the opposite at the Br6 stage (Fig. 3A). Moreover, we identified 515 and 512 DAS in *Nr*-MG vs AC-MG and *Nr*-Br6 vs AC-Br6, respectively (Table S13). In the *Nr*-MG vs AC-MG, 490 DAS were located on 464 transcripts, with 210 sites on 206 transcripts showing upregulated acetylation modification and 280 sites on 274 transcripts showing downregulated modification (Table S14), the ac^4^C sites with low acetylation levels were more abundant than those with high acetylation levels, indicating a general decrease in RNA (ac^4^C) acetylation modification in *Nr* mutant fruit compared to AC fruit at the MG stage. In contrast to the findings at the MG stage, in the *Nr*-Br6 vs AC-Br6, 479 DAS were located on 454 transcripts, with 261 sites on 253 transcripts showing upregulated acetylation modification and 218 sites on 214 transcripts showing downregulated acetylation (Table S14), the ac^4^C sites with high acetylation levels were more abundant than those with low acetylation levels, indicating a general increase in mRNA (ac^4^C) acetylation modification in the *Nr* mutant fruit compared to AC fruit at the Br6 stage.

**Fig. 6:**
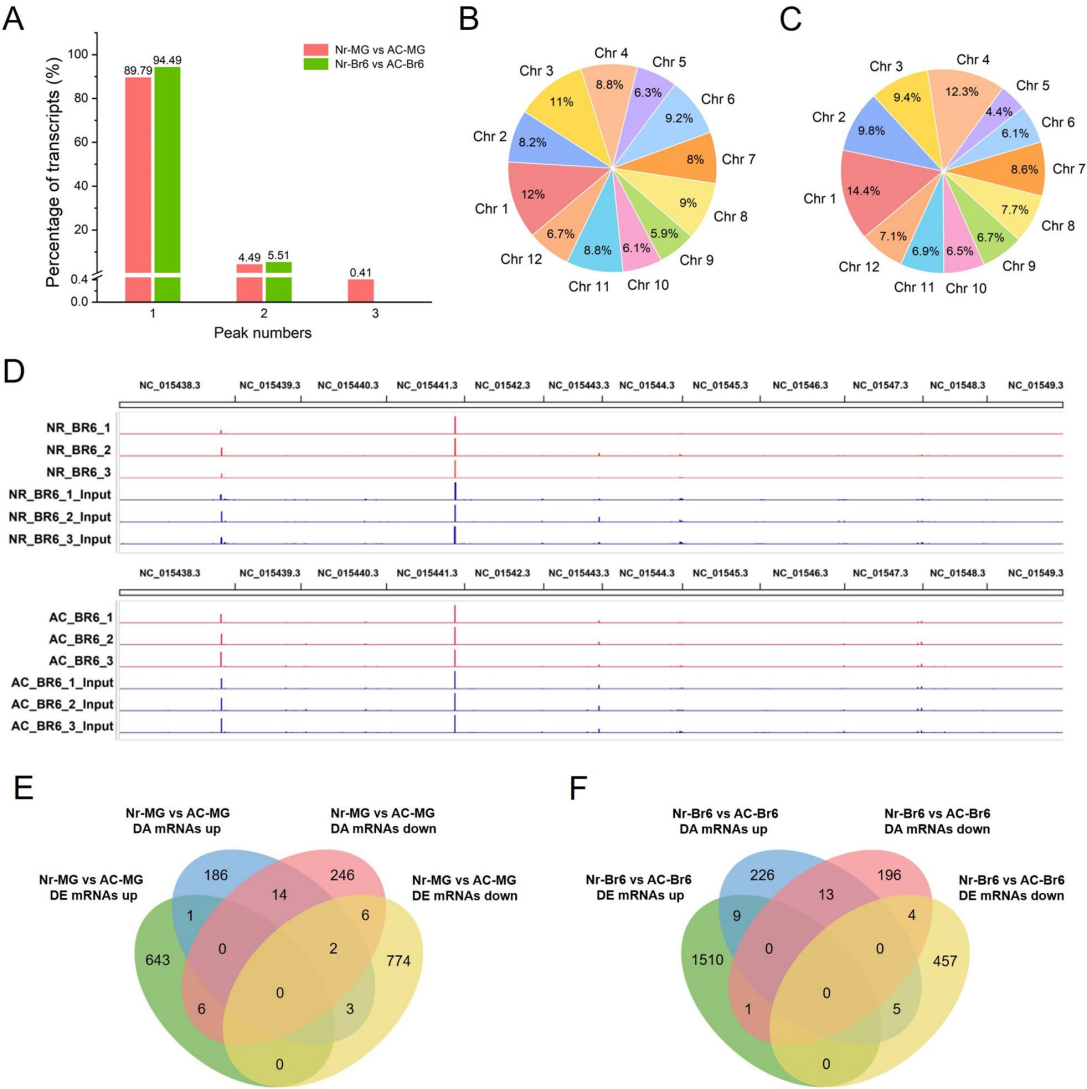
Overview of ac^4^C acetylation profiles in *Nr* vs AC tomatoes at the MG and Br6 stages. **A** Proportions of the ac^4^C-modified transcripts containing different ac^4^C peak numbers in *Nr*-MG vs AC-MG and *Nr*-Br6 vs AC-Br6. **B** The pie chart presenting the distribution proportion of differential ac^4^C modification within mRNA across the 12 chromosomes in *Nr*-MG vs AC-MG. **C** The distribution proportion of differential ac^4^C modification within mRNA across the 12 chromosomes in *Nr*-Br6 vs AC-Br6. **D** Integrated Genome Viewer (IGV) snapshots showing differences in mRNA acetylation (ac^4^C) between two types of tomato fruit at the Br6 stage. **E** Correlation analysis of gene expression and mRNA acetylation (ac^4^C) in *Nr*-MG vs AC-MG. **F** Correlation analysis of gene expression and mRNA acetylation (ac^4^C) in *Nr*-Br6 vs AC-Br6

In the comparison group *Nr*-MG vs AC-MG, among gene transcripts containing differential ac^4^C modification, the majority (440, 89.79%) contained one ac^4^C peak, 22 transcripts (4.49%) displayed two ac^4^C peaks, and only 2 transcripts (0.41%) exhibited three ac^4^C peaks (Fig. 6A, Table S14). However, at the Br6 stage, the majority (94.49%) contained one ac^4^C peak, and 5.51% displayed two ac^4^C peaks (Fig. 6A, Table S14). Moreover, the distribution of DAS was broad across all chromosomes of the tomato genome at the MG and Br6 stage (Fig. 6B, C). The differential distribution of ac^4^C acetylation modification between *Nr* and AC tomatoes at the Br6 and MG stage were shown in Fig. 6D and Figure S8, respectively. In the *Nr*-MG vs AC-MG, the majority of the DAS (436, 89%) were mainly located within the CDS, with fewer ac^4^C sites (44, 9%) in the 3'UTR, and only a few ac^4^C sites (10, 2%) in the 5'UTR, while no ac^4^C sites were found in the intron region (Table S14). In the *Nr*-Br6 vs AC-Br6, the majority of the DAS were mainly located within the CDS (427, 89.1%), with a few ac^4^C sites in the 3'UTR (34, 7.1%) and 5'UTR (16, 3.3%), and only 2 ac^4^C sites were located in the intron region (Table S14).

### The integrated analysis of RNA-seq and ac^4^C-seq data at the MG and Br6 stage in AC and *Nr* tomato fruits

The combined analysis of DA mRNAs and DE mRNAs was performed. A total of 18 mRNAs exhibited differences in both ac^4^C acetylation modification levels and expression levels in *Nr*-MG vs AC-MG, of which 6 mRNAs displayed higher ac^4^C levels, while 14 mRNAs exhibited lower ac^4^C acetylation levels (Fig. 6E, Table S15). Interestingly, both the gene (Solyc06g053710.3) encoding ETR and the gene (Solyc11g017470.2) encoding NAC4 domain protein have two different mRNA ac^4^C sites with significantly different acetylation modification levels.

Notably, at the MG stage, the ac^4^C sites on DE mRNAs *ACO* (Solyc02g036350.3) and *ETR* (Solyc06g053710.3) with reduced expression displayed hypoacetylation (Table S15). Interestingly, the ac^4^C acetylation levels at different sites within the same mRNA *ETR* (Solyc06g053710.3) were found to be different (Table S15), suggesting the complex regulatory effects of acetylation modifications at different sites of mRNAs on gene expression.

A total of 19 mRNAs exhibited differences in both ac^4^C acetylation modification level and expression levels in *Nr*-Br6 vs AC-Br6 (Fig. 6F, Table S15). Among the 14 mRNAs with higher ac^4^C acetylation levels, 9 mRNAs showed higher expression levels (Fig. 6F, Table S15). On the other hand, among the 5 mRNAs with lower ac^4^C acetylation levels, 4 mRNAs showed lower expression levels (Fig. 6F, Table S15). Overall, these results indicated that ac^4^C acetylation modification may have a positive regulatory effect on gene expression.

## Discussion

The tomato fruit ripening process is dynamically regulated by gene expression and influenced by transcriptional and post-transcriptional regulation (Ecker 2013; Lang et al. 2017; Jia et al. 2022; Li et al. 2022). Compared with wild-type tomato, *Nr* tomato (*Never ripe*), an ethylene *SlETR3* receptor loss-of-function mutant, is insensitive to ethylene and shows incomplete ripening (Nascimento et al. 2021). Given the important role of the plant hormone ethylene in fruit ripening (Fenn and Giovannoni 2021; Li et al. 2021), we focused on the expression changes of ethylene related genes during the ripening process of tomato fruit from both genotypes. Previous studies have shown that compared to wild-type tomato fruit, the genes *ACSs*, *ACOs*, and *ERFs* were downregulated in the *rin* mutant fruit (Fujisawa et al. 2012; Kumar et al. 2012; Qin et al. 2012; Kumar et al. 2016), which is similar to our results. Compared to AC fruit, the expression levels of many other ethylene-related genes *ACS*, *ACO*, *Nr*, *green ripe like 1*, *ETRs* and *ERFs* were reduced in *Nr* fruit at the MG stage (Table S16); for tomatoes at six days after the breaker stage (Br6), the expression of *ACS2*, *ACO1*, *ETRs*, and *ERFs* was lower in *Nr* fruit than in AC fruit, while the expression of *CTR1* and other *ERFs* were higher in *Nr* tomato (Table S16). These results explained why ethylene insensitive *Nr* tomato cannot mature normally (Lanahan et al. 1994).

Due to damage to the ethylene signaling pathway in ethylene insensitive mutants *Nr* tomato, the color and texture of *Nr* tomato also undergo a series of changes (Kou et al. 2021). The change in color is the most representative characteristic of tomato fruit ripening. Mature *Nr* mutant fruit accumulate very little lycopene, and remain yellow instead of red at the Br6 stage (Yen et al. 1995; Carvalho et al. 2011). During tomato fruit ripening, we observed strong up regulation of *PSY* and *CRTISO* in *Nr* fruit compared to small up regulation in AC fruit, whereas *ZDS* and *BCH* exhibited small upregulation, and *LCYE* showed a small downregulation in *Nr* fruit compared to strong up and down regulation in AC fruit (Table S7). These results are consistent with previous analysis (Wang et al. 2009; Karlova et al. 2011). The changes in texture during tomato fruit ripening are usually caused by upregulation of cell wall modifying enzymes (Fry 2004; Brummell 2006; Irfan et al. 2016, Shi et al. 2022; Shi et al. 2023). We found that at the Br6 stage, two *PGs*, one *PE*, and two *PAEs* genes were downregulated in the *Nr* mutant compared to AC tomato (Table S17), and *Nr* tomatoes remain notably firmer than AC tomato for months. Similarly, genes involved in cell wall modification exhibit differential expression in the *rin* mutant compared to the wild-type, such as *β-Gal*, *PG*, *β-Glu*, *PAE*, *PL*, *PE*, *EXP*, etc. (Fujisawa et al. 2012; Kumar et al. 2012; Qin et al. 2012; Kumar et al. 2016). Moreover, lncRNAs targeting these key genes related to fruit ripening exhibited similar expression patterns and fold change to theirs targets (Table S8), and they play significant roles in tomato fruit ripening by positively regulating gene expression.

ac^4^C is an ancient and highly conserved RNA modification present in all life forms (Sas-Chen et al. 2020). While its existence in tRNA and rRNA has long been established, its presence within mRNA in plants has remained largely unknown. Recent studies have confirmed the existence of ac^4^C, an enzymatic modification, on mRNA in plants, including model plants Arabidopsis and rice (Li et al. 2023; Wang et al. 2023). In Arabidopsis, only 0.115% of cytidine nucleosides in total RNA were found to be N^4^-acetylated, which is much lower than in human cells (Arango et al. 2018). Our study identified a total of 29,211 RNAs (mRNAs and lncRNAs) in four tomato samples (Table S18), with 1,211 mRNAs showing ac^4^C acetylation modification (Table S19). However, no acetylation modification was detected on lncRNAs (Table S19). Compared to the 470 ac^4^C peaks identified in Arabidopsis, we identified a total of 1,414 ac^4^C peaks in the four tomato samples (Table S19). Additionally, our findings showed that ac^4^C modifications are mainly located in the CDS region of mRNAs, with significant enrichment in the 3' UTR region (Fig. 3G, H, Table S19), consistent with the distribution in Arabidopsis (Wang et al. 2023). These results suggest that ac^4^C modification may have similar biological functions in plants.

Acetylation motifs have been successively proven to exist in mammals and plants. Arango et al. (2018) identified a conserved 29-nt repeating CXX motif in mammals. Subsequently, Wang et al. (2023) identified a significantly enriched C-rich motif CCWCCDCC in Arabidopsis plants. In addition, another study has demonstrated the existence of a enriched "CCAA" motif in ac^4^C peaks common to both Arabidopsis and rice (Li et al. 2023). Similar to these studies, we identified numerous motifs enriched in ac^4^C peaks using HOMER in tomato fruits. Notably, we found a significant C-rich motif TGCCCCGATC in AC-Br6 fruits (Fig. 4C), indicating that the enriched motifs in ac^4^C peaks in tomatoes and other plants may have potential common characteristics.

**Fig. 7:**
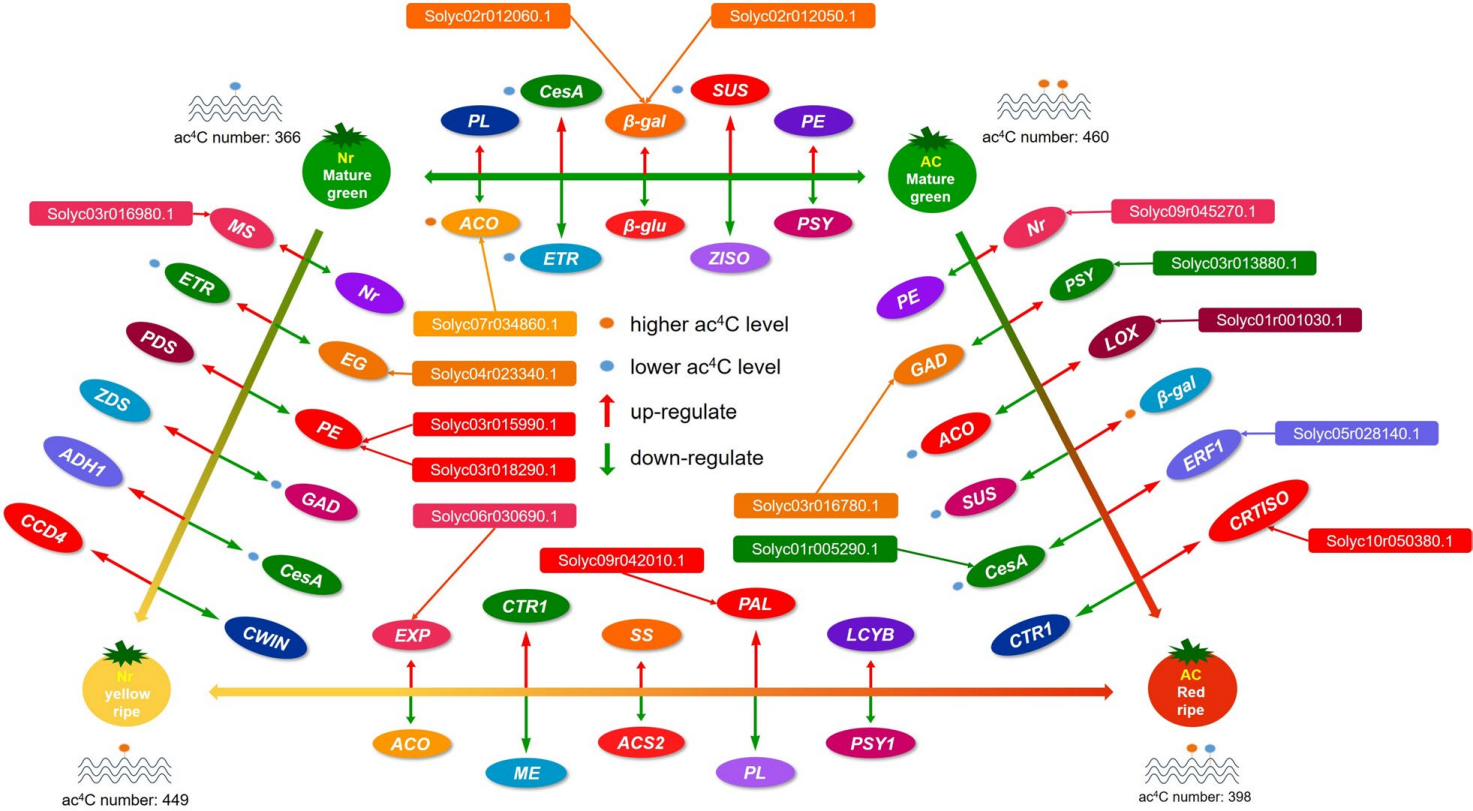
The network model of genes potentially modulated by lncRNA and acetylation modification that regulate the ripening process of AC and *Nr* tomato fruits. The box represents lncRNA, the ellipse represents genes, the small orange and blue ellipses represent high and low acetylation, respectively, and the red and green arrows represent up regulation and down regulation, respectively

RNA modifications have been shown to impact RNA metabolic activity, ultimately affecting RNA's biological function by altering stability, splicing, localization, and translation processes (Gilbert et al. 2016; Roundtree et al. 2017; Frye et al. 2018). Studies on RNA acetylation modification have demonstrated that ac^4^C modification in mRNAs can enhance RNA stability, both in mammals and plants (Arango et al. 2018; Li et al. 2023), although the strength of this modification may vary among different species. Our research further supports this viewpoint, we found that the amounts of mRNA acetylation vary between different varieties types or mature stages in tomato fruits (Fig. 3A), which is similar to the situation of mRNA methylation in mammals and plants (Zhong et al. 2008; Meyer et al. 2012). Specifically, the number of acetylation, and the acetylation modification levels on most transcripts decreased during the ripening process in AC fruits (Fig. 3A, Table S10). In the downregulated mRNAs, the acetylation levels of genes related to fruit ripening, *CesA*, *ACO*, and *SUS*, decreased by about 12, 2, and 3 times, respectively (Table S11). The corresponding gene expression levels decreased by 3, 10, and 6.5 times (Table S11), indicating a possible positive correlation between the acetylation levels and the mRNA stability. On the contrary, the number of acetylation site and the acetylation levels on most mRNAs increased during the ripening process of *Nr* fruits (Fig. 3A, Table S10). In the upregulated mRNA, the acetylation levels of *ethylene-responsive transcription factor ABR1-like* and *AMY* increased by approximately 2.6 and 4.4 times, respectively, and their expression levels also increased by 5.4 and 2.1 times, respectively (Table S11). Nevertheless, we also found different changes in acetylation levels and expression levels on certain genes, such as a decrease in acetylation levels on *ACO* and *ETR*, but an upward trend in expression levels (Table S11), consistent with the findings of Wang et al. (2023). Therefore, the exact relationship between acetylation modification levels and transcript abundance is largely unknown, further investigation into the molecular mechanisms behind this phenomenon presents an interesting avenue for future research. Based on the above results, a gene regulatory network model was constructed to demonstrate the comprehensive impact of mRNA, lncRNA, and acetylation modification on fruit ripening process of tomato fruits (Fig. 7).

As is well known, the *Nr* mutation in AC results in only partial ethylene insensitivity (Hobson 1967). Study has shown that the tomato *Nr* mutant exhibits demonstrable sensitivity to ethylene in the presence of as little as 1 ppm of ethylene, suggested that the partial ripening observed in mature *Nr* fruit may be due to partial ethylene responsiveness (Yen et al. 1995). Therefore, the *Nr* fruit in the Ailsa Craig background ripen to a greater extent than those in the Pearson background, because they retain residual ethylene responsiveness (Lanahan et al. 1994; Yen et al. 1995). Consistent with our research findings, we observed partially ripe fruit in the *Nr* mutant at Br6, while more fully ripe fruit was observed in the AC-Br6, indicating that *Nr*-Br6 can be viewed as an intermediate ethylene phenotype between AC-MG and AC-Br6, and that this is consistent with the ripening-associated nature of ac^4^C acetylation. Interestingly, we have found that the expression of most DEGs decreased with tomato fruit ripening, but these DEGs exhibited higher expression in *Nr* fruit compared with AC tomato at the Br6 stage. Correspondingly, at the Br6 stage, the overall mRNA acetylation level in *Nr* fruit is higher than that of AC fruit. These results indicated that ac^4^C acetylation may be ripening related and in track with many ripening genes at the Br6 stage.

In conclusion, this study utilized the ac^4^C-seq method to confirm the existence of ac^4^C acetylation modification on mRNA in the model plant tomato. By comparing the changes in ac^4^C acetylation levels in two tomato fruit at different maturity stages, we demonstrated that ac^4^C modification changes mediated by ethylene signaling may play a regulatory role in tomato fruit ripening. During fruit ripening, acetylation levels exhibited varying trends in different tomato varieties, suggesting the acetylation modification level was affected in *Nr* mutant. The integrated analysis suggested that acetylation modification may positively regulate gene expression, though further investigation is needed to elucidate its mechanism of action. These findings provide valuable insights into distinct acetylation modification events during the ripening process in wild-type and mutant tomato fruit for the first time.

## Materials and methods

### Plant materials

Fruit of two tomato varieties, the WT tomato (AC) and homozygous ethylene receptor mutant tomato (*Nr*) in the Ailsa Craig (AC) background, were grown in a growth chamber under standard cultivation conditions (24 °C, relative humidity of 75%, 16 h/8 h light/dark photoperiod) at the Zhejiang Academy of Agricultural Sciences. They were harvested at two different maturity stages: the MG stage (mature green, approximately 39 d after anthesis for both types) and RR stage (red ripening, 6 d after the BR (breaker) stage), at which point the *Nr* mutant exhibited an yellow phenotype. Fruit of each cultivar with uniform size and without diseases and visual blemishes were picked and divided into two groups : AC and *Nr* mutant fruit, then transferred to the laboratory as soon as possible. Fruit at two different maturity stages were sampled for each group, pericarp tissue of two cultivars' tomatoes fruit were cut into 3−5 mm pieces and collected and frozen in liquid nitrogen and stored at −80 °C for subsequent assay. There are three repetitions for per stage, and 12 fruit for per replication.

### UID RNA-seq

The experiment involving UID RNA-seq and high-throughput sequencing, as well as data analysis, were carried out by Seqhealth Technology Co., LTD (Wuhan, China).

### RNA extraction, library preparation and sequencing

The methods by Chomczynski et al. were followed to extract total RNAs from pericarp tissue of AC and *Nr* tomato fruit using TRIzol Reagent (Invitrogen, cat. NO 15596026) (Qin et al. 2022). RNA quality was determined by examining A260/A280 with NanodropTM OneCspectrophotometer (Thermo Fisher Scientific Inc) (Zhu et al. 2021). RNA Integrity was confirmed by 1.5% agarose gel electrophoresis (Zhu et al. 2021). Qualified RNAs were finally quantified by Qubit3.0 with QubitTM RNA Broad Range Assay kit (Life Technologies, Q10210) (Zhu et al. 2021).

2 μg total RNAs were used for stranded RNA sequencing library preparation using KC-DigitalTM Stranded mRNA Library Prep Kit for Illumina® (Catalog NO. DR08502, Wuhan Seqhealth Co., Ltd. China) following the manufacturer’s instruction (Zhu et al. 2021). The kit eliminates duplication bias in PCR and sequencing steps, by using unique molecular identifier (UMI) of 8 random bases to label the pre-amplified cDNA molecules (Zhu et al. 2021). The library products corresponding to 200-500 bps were enriched, quantified and finally sequenced on DNBSEQ-T7 sequencer (MGI Tech Co., Ltd. China) with PE150 model (Zhu et al. 2021).

### RNA-seq data analysis

Raw sequencing data was first filtered by Trimmomatic (version 0.36), low-quality reads were discarded and the reads contaminated with adaptor sequences were trimmed (Yang et al. 2023). Clean Reads were further treated with in-house scripts to eliminate duplication bias introduced in library preparation and sequencing (Yang et al. 2023). In brief, clean reads were first clustered according to the UMI sequences, in which reads with the same UMI sequence were grouped into the same cluster (Yang et al. 2023). Reads in the same cluster were compared to each other by pairwise alignment, and then reads with sequence identity over 95% were extracted to a new sub-cluster (Yang et al. 2023). After all sub-clusters were generated, multiple sequence alignment was performed to get one consensus sequence for each sub-clusters (Yang et al. 2023). After these steps, any errors and biases introduced by PCR amplification or sequencing were eliminated (Yang et al. 2023).

The de-duplicated consensus sequences were used for standard RNA-seq analysis (Zhu et al. 2021). They were mapped to the reference genome of *Solanum lycopersicum* from https://solgenomics.net/ftp/tomato_genome/annotation/ITAG4.0_release/ using STAR software (version 2.5.3a) with default parameters. Reads mapped to the exon regions of each gene were counted by featureCounts (Subread-1.5.1; Bioconductor) and then RPKM was calculated (Yousaf et al. 2022). Genes differentially expressed between groups were identified using the edgeR package (version 3.12.1) (Yousaf et al. 2022). A p-value cutoff of 0.05 and Fold-change cutoff of 2 were used to judge the statistical significance of gene expression differences (Yousaf et al. 2022). Gene ontology (GO) analysis and Kyoto encyclopedia of genes and genomes (KEGG) enrichment analysis for differentially expressed genes were both implemented by KOBAS software (version: 2.1.1) with a P-value cutoff of 0.05 to judge statistically significant enrichment (Yousaf et al. 2022). Alternative splicing events were detected by using rMATS (version 3.2.5) with a FDR value cutoff of 0.05 and an absolute value of Δψ of 0.05 (Yousaf et al. 2022).

### ac^4^C-seq

ac^4^C-seq experiment and high through-put sequencing and data analysis were conducted by Seqhealth Technology Co., LTD (Wuhan, China).

### RNA extraction, library preparation and sequencing

Total RNA was extracted from AC and *Nr* tomto fruit using TRIzol Reagent (Invitrogen, cat. NO 15596026) following the methods by Chomczynski et al (Qin et al. 2022). DNA digestion was carried out after RNA extraction by DNaseI (Yousaf et al. 2022). RNA quality was determined by examining A260/A280 with NanodropTM OneCspectrophotometer (Thermo Fisher Scientific Inc) (Yousaf et al. 2022). RNA Integrity was confirmed by Qsep100 (bioptic) or Agilent5300 (Agilent) (Yousaf et al. 2022).

30 μg total RNA was used for mRNA capture using KAPA mRNA Capture Kit (KK8441) following the manufacturer’s instruction (Yousaf et al. 2022). Take a small amount of captured RNA as Input, and the remaining RNA was treated with NaCNBH3 at 25℃ for 20 minutes; RNA clean beads were used to retrieve the treated RNA (Yousaf et al. 2022). Then, KC Digital Stranded mRNA Library Prep Kit for Illumina (DR085-02) was used for library construction (Yousaf et al. 2022). The kit eliminates duplication bias in PCR and sequencing steps, by using unique molecular identifier (UMI) of 8 random bases to label the pre-amplified cDNA molecules (Yousaf et al. 2022). The library products corresponding to 200-500 bps were enriched, quantified and finally sequenced on MGISEQ-T7 (MGI) with PE150 model (Yousaf et al. 2022).

### Data analysis

Raw sequencing data were first filtered by fastp (version 0.23.1), low-quality reads were discarded and the reads contaminated with adaptor sequences were trimmed (Wei et al. 2023). UMI (version 1.0), the self-developed software of Seqhealth Co., Ltd were used to eliminate duplication. Reads after duplication removal were mapped to the reference genome of *Solanum lycopersicum* from https://solgenomics.net/ftp/tomato_genome/annotation/ITAG4.0_release/ using STAR (version 2.7.6a) with default parameters. Sambamba (version 0.7.1) was used for sam/bam format conversion and index build. The featureCounts (version 1.5.1) was used for reads distribution statistic. The JACUSA software (Version 2.0.1) was used for detecting SNV sites and obtaining the corresponding pileup. Self-developed software Rigel (Version 1.0.0) was used to performing statistical test on detected SNV sites, and identifying DAS. The Homer (version 4.10) was used for motifs analysis (Wei et al. 2023). Gene ontology (GO) analysis and Kyoto encyclopedia of genes and genomes (KEGG) enrichment analysis for annotated genes were both implemented by KOBAS software (version: 2.1.1) with a corrected P-value cutoff of 0.05 to judge statistically significant enrichment (Wei et al. 2023).

### Supplementary Information

**Additional file 1:** **Table S1.** The differentially expressed genes (DEGs) identified in AC-Br6 vs AC-MG, *Nr*-Br6 vs *Nr*-MG, *Nr*-MG vs AC-MG and *Nr*-Br6 vs AC-Br6. **Table S2.** The differentially expressed (DE) lncRNAs in AC-Br6 vs AC-MG, *Nr*-Br6 vs *Nr*-MG, *Nr*-MG vs AC-MG and *Nr*-Br6 vs AC-Br6. **Table S3.** The DE lncRNAs and corresponding DE target genes identified in AC-Br6 vs AC-MG, *Nr*-Br6 vs *Nr*-MG, *Nr*-MG vs AC-MG and *Nr*-Br6 vs AC-Br6. **Table S4.** The DEGs in the overlap of between *Nr*-Br6 vs *Nr*-MG and AC-Br6 vs AC-MG. **Table S5.** The DE lncRNAs in the overlap of between *Nr*-Br6 vs *Nr*-MG and AC-Br6 vs AC-MG. **Table S6.** The common DE lncRNAs and DE target genes during the ripening process of *Nr* and AC tomatoes. **Table S7.** The DEGs related to fruit ripening in the overlap of between *Nr*-Br6 vs *Nr*-MG and AC-Br6 vs AC-MG. **Table S8.** The common DE lncRNAs and DE target genes related to fruit ripening in *Nr* and AC tomatoes. **Table S9.** The DAS identified in AC-Br6 vs AC-MG and *Nr*-Br6 vs *Nr*-MG. **Table S10.** The DAS on transcripts identified in AC-Br6 vs AC-MG and *Nr*-Br6 vs *Nr*-MG. **Table S11.** The intersection of differentially acetylated mRNAs (DA mRNAs) and differentially expressed mRNAs (DE mRNAs) in AC-Br6 vs AC-MG and *Nr*-Br6 vs *Nr*-MG. **Table S12.** The intersection of DA mRNAs and DE mRNAs in the overlap of *Nr*-Br6 vs *Nr*-MG and AC-Br6 vs AC-MG. **Table S13.** The DAS identified in *Nr*-MG vs AC-MG and *Nr*-Br6 vs AC-Br6. **Table S14.** The DAS on transcripts identified in *Nr*-MG vs AC-MG and *Nr*-Br6 vs AC-Br6. **Table S15.** The intersection of DA mRNAs and DE mRNAs in *Nr*-MG vs AC-MG and *Nr*-Br6 vs AC-Br6. **Table S16.** The DEGs related to plant hormones synthesis and signal transduction in *Nr*-MG vs AC-MG and *Nr*-Br6 vs AC-Br6. **Table S17.** The DEGs related to fruit texture in *Nr*-Br6 vs AC-Br6. **Table S18.** The RNA (mRNA and lncRNA) identified in four tomato samples. **Table S19.** Transcripts containing acetylation modification sites.

**Additional file 2:** **Figure S1.** The KEGG pathway enrichment of upregulated DEGs in AC-Br6 vs AC-MG and *Nr*-Br6 vs *Nr*-MG. **Figure S2.** The KEGG pathway enrichment of DEGs in Nr-MG vs AC-MG. **Figure S3.** The KEGG pathway enrichment of DEGs in* Nr*-Br6 vs AC-Br6. **Figure S4.** The KEGG pathway enrichment of target genes of DE lncRNAs in AC-Br6 vs AC-MG. **Figure S5.** The KEGG pathway enrichment of target genes of DE lncRNAs in *Nr*-Br6 vs *Nr*-MG. **Figure S6.** The KEGG pathway enrichment of target genes for DE lncRNAs in the *Nr*-MG vs AC-MG. **Figure S7.** The KEGG pathway enrichment of target genes for DE lncRNAs in the *Nr*-Br6 vs AC-Br6. **Figure S8.** Integrated Genome Viewer (IGV) snapshots showing differences in mRNA acetylation (ac^4^C) between fruits from both genotypes at the MG stage.

## Data Availability

The datasets used and/or analysed during the current study are available from the corresponding author on reasonable request.

## Abbreviations

ac^4^C: N^4^-acetylcytidine
UID: Unique Identifier
Nr: Never-ripe
ACO: 1-aminocyclopropane-1-carboxylate oxidase
ACS: 1-aminocyclopropane-1-carboxylate synthase
ETR: Ethylene receptor
ERF: Ethylene response factor
PSY: Phytoene synthase
ZISO: 15-cis-zeta-carotene isomerase
β-Glu: Beta-glucosidase
EG: Endoglucanase
PL: Pectate lyase
PAE: Pectin acetylesterase
PG: Polygalacturonase
PE: Pectinesterase
MDH: malate dehydrogenase
ME: Malic enzyme
LOX: Lipoxygenase
AAT: Alcohol acyl transferase
PAL: Phenylalanine ammonia-lyase
GAD: Glutamate decarboxylase
CesA: Cellulose synthase
AMY: Alpha-amylase
β-Gal: Beta-galactosidase
SUS: Sucrose synthase
EXP: Expansin
SS: Starch synthase
ADH: Alcohol dehydrogenase
SPS: Sucrose-phosphate synthase
STP: Sugar transporter
HK: Hexokinase
CWIN: Cell-wall invertase
BMY: Beta-amylase
DAS: Differential ac^4^C sites
