# Supplementary material for: Dissection of mRNA ac4C acetylation modifications in AC and Nr fruits: insights into the regulation of fruit ripening by ethylene
Source: Mol Hortic. 2024 Feb 19;4:5. doi: 10.1186/s43897-024-00082-7 (PMC10875755; doi:10.1186/s43897-024-00082-7)
Supplement: Supplementary file 2 — Additional file 2: Figure S1. The KEGG pathway enrichment of upregulated DEGs in AC-Br6 vs AC-MG and Nr-Br6 vs Nr-MG. Figure S2. The KEGG pathway enrichment of DEGs in Nr-MG vs AC-MG. Figure S3. The KEGG pathway enrichment of DEGs in Nr-Br6 vs AC-Br6. Figure S4. The KEGG pathway enrichment of target genes of DE lncRNAs in AC-Br6 vs AC-MG. Figure S5. The KEGG pathway enrichment of target genes of DE lncRNAs in Nr-Br6 vs Nr-MG. Figure S6. The KEGG pathway enrichment of target genes for DE lncRNAs in the Nr-MG vs AC-MG. Figure S7. The KEGG pathway enrichment of target genes for DE lncRNAs in the Nr-Br6 vs AC-Br6. Figure S8. Integrated Genome Viewer (IGV) snapshots showing differences in mRNA acetylation (ac4C) between fruits from both genotypes at the MG stage. [file 43897_2024_82_MOESM2_ESM.docx]

**Supplementary Figures**

**Figure S1**


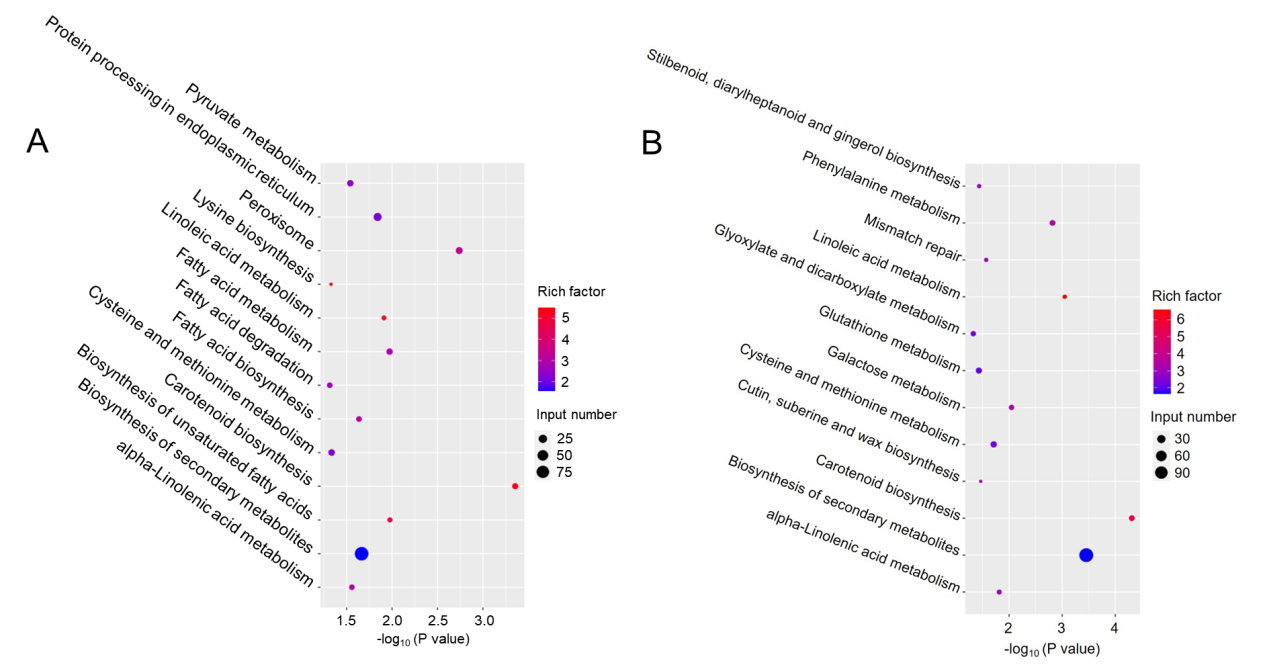


**Figure S1. A** The KEGG pathway enrichment of upregulated DEGs in AC-Br6 vs AC-MG. **B** The KEGG pathway enrichment of upregulated DEGs in *Nr*-Br6 vs *Nr*-MG.

**Figure S2**


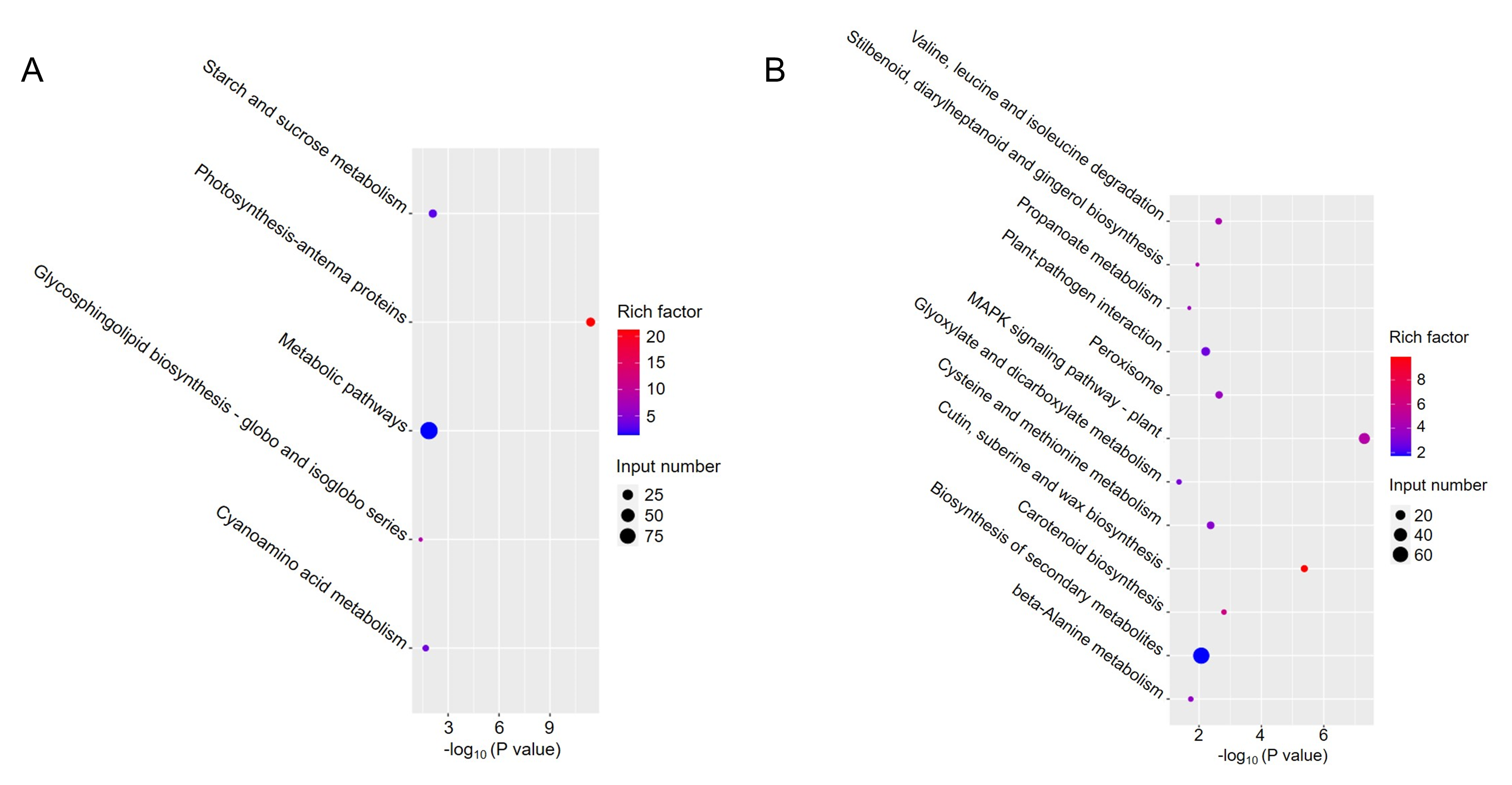


**Figure S2. A** The KEGG pathway enrichment of upregulated DEGs in *Nr*-MG vs AC-MG. **B** The KEGG pathway enrichment of downregulated DEGs in *Nr*-MG vs AC-MG.

**Figure S3**


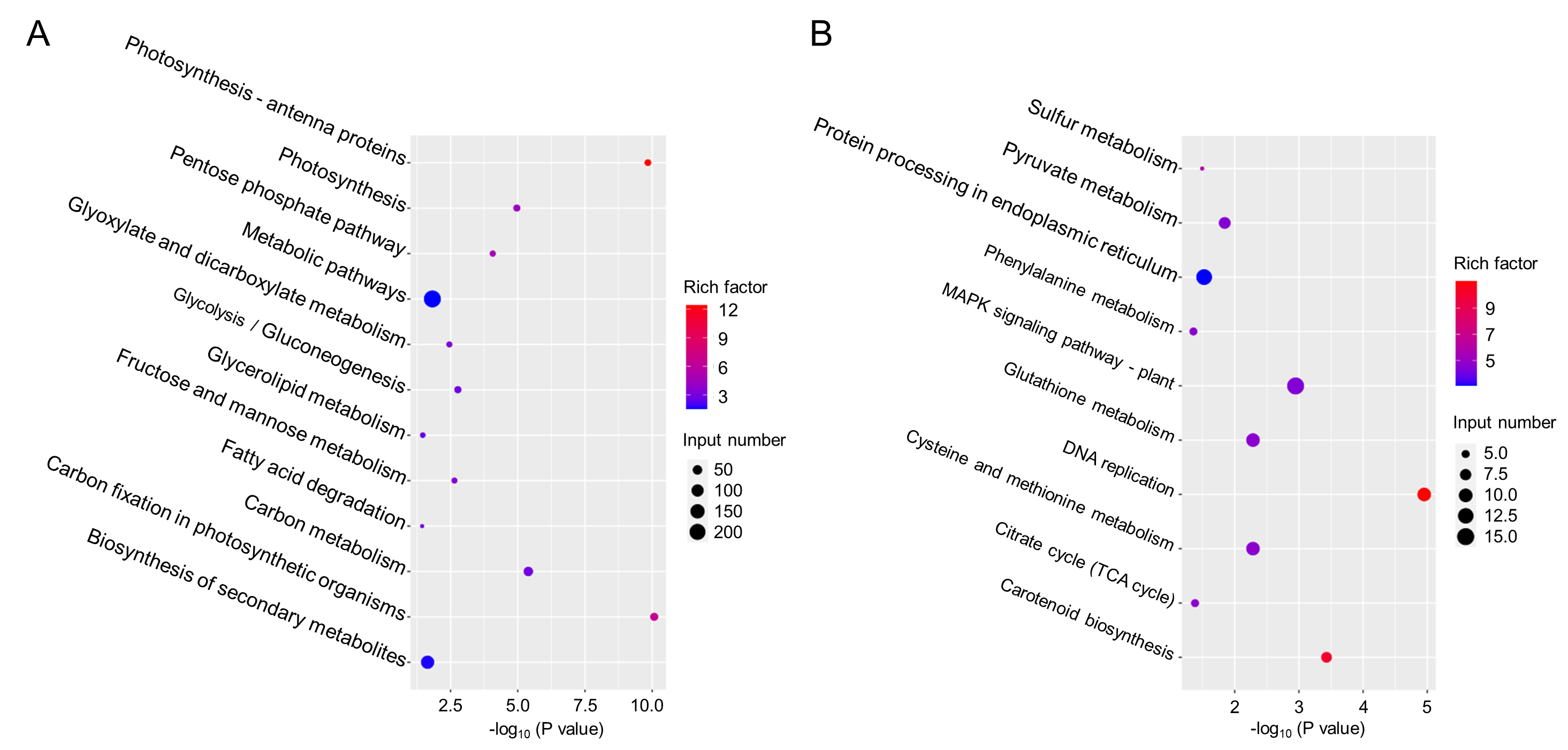


**Figure S3. A** The KEGG pathway enrichment of upregulated DEGs in *Nr*-Br6 vs AC-Br6. **B** The KEGG pathway enrichment of downregulated DEGs in *Nr*-Br6 vs AC-Br6.

**Figure S4**


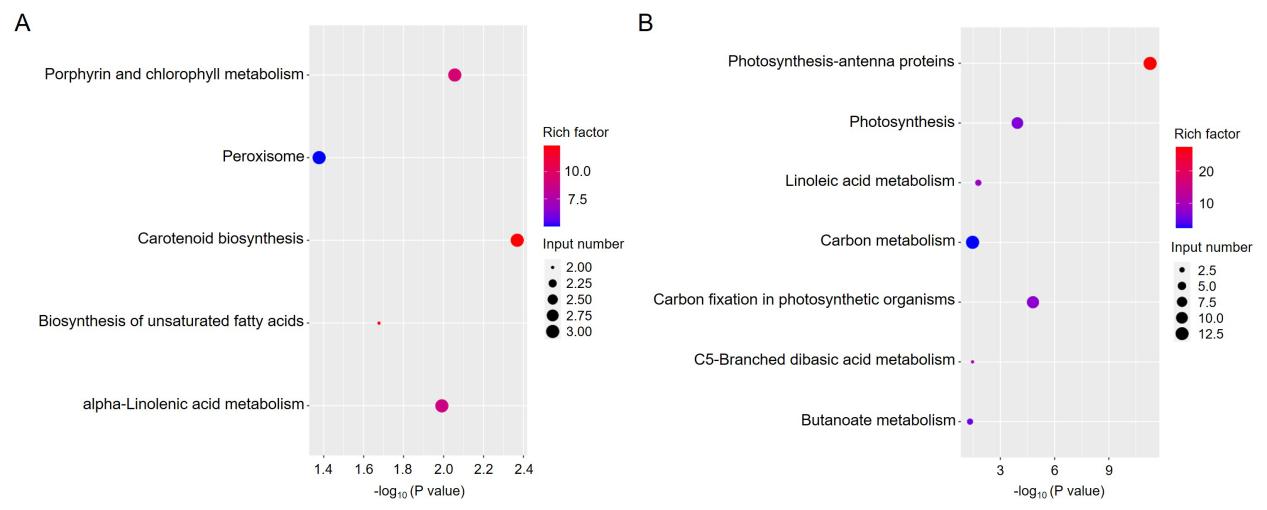


**Figure S4. A** The KEGG pathway enrichment of target genes of upregulated DE lncRNAs in AC-Br6 vs AC-MG. **B** The KEGG pathway enrichment of target genes of downregulated DE lncRNAs in AC-Br6 vs AC-MG.

**Figure S5**

**
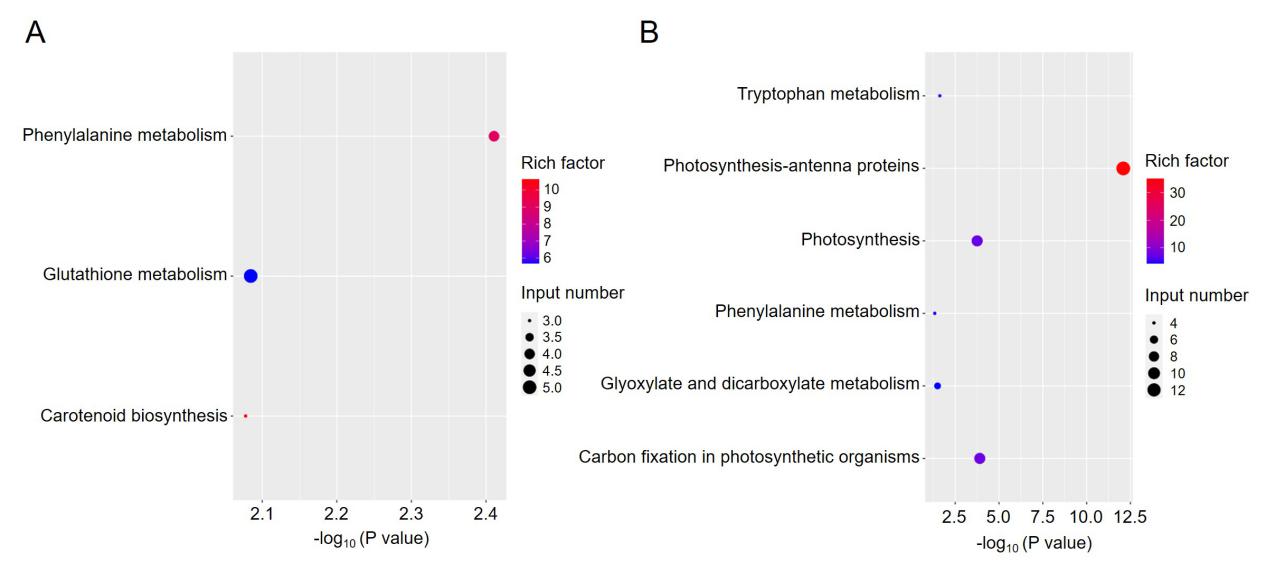
**

**Figure S5. A** The KEGG pathway enrichment of target genes of upregulated DE lncRNAs in *Nr*-Br6 vs *Nr*-MG. **B** The KEGG pathway enrichment of target genes of downregulated DE lncRNAs in *Nr*-Br6 vs *Nr*-MG.

**Figure S6**


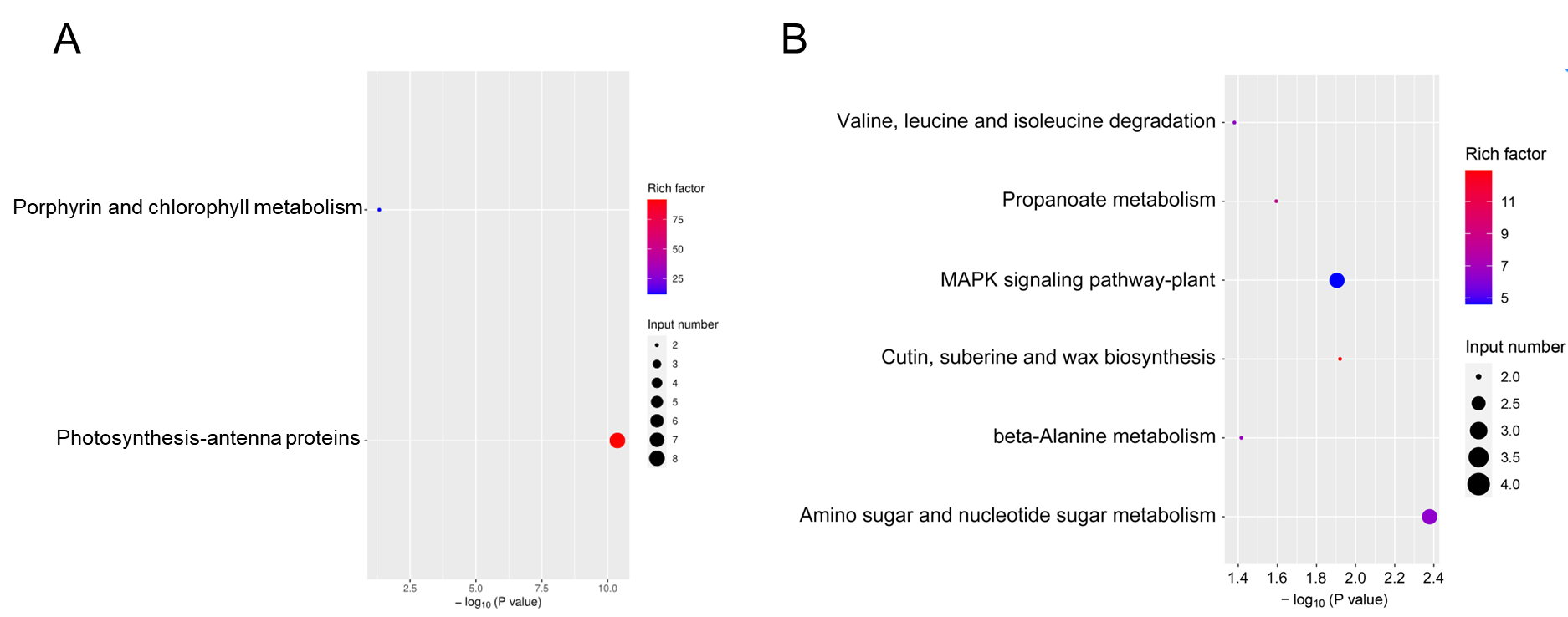


**Figure S6. A** The KEGG pathway enrichment of target genes for upregulated DE lncRNAs in the *Nr*-MG vs AC-MG. **B** The KEGG pathway enrichment of target genes for downregulated DE lncRNAs in the *Nr*-MG vs AC-MG.

**Figure S7**


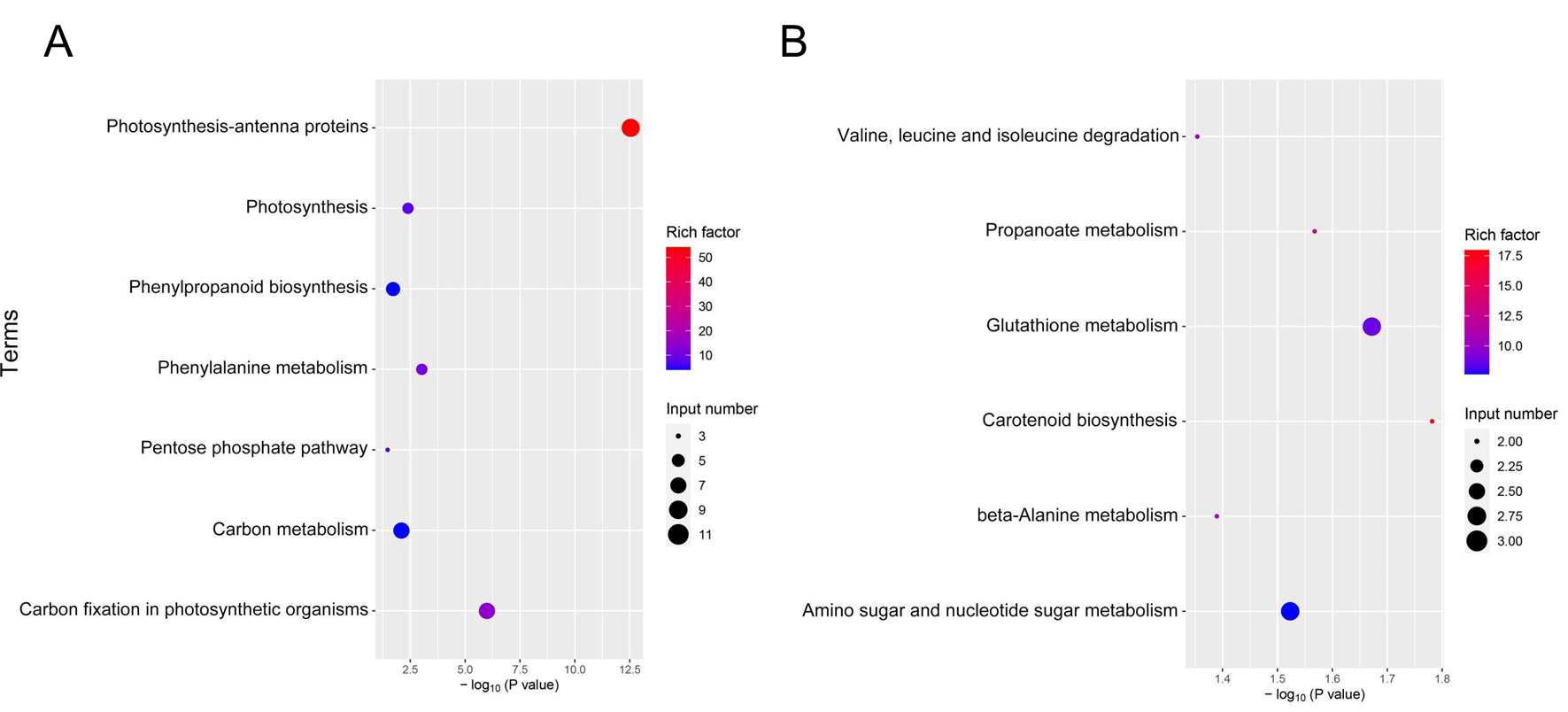


**Figure S7. A** The KEGG pathway enrichment of target genes for upregulated DE lncRNAs in the *Nr*-Br6 vs AC-Br6. **B** The KEGG pathway enrichment of target genes for downregulated DE lncRNAs in the *Nr*-Br6 vs AC-Br6.

**Figure S8**


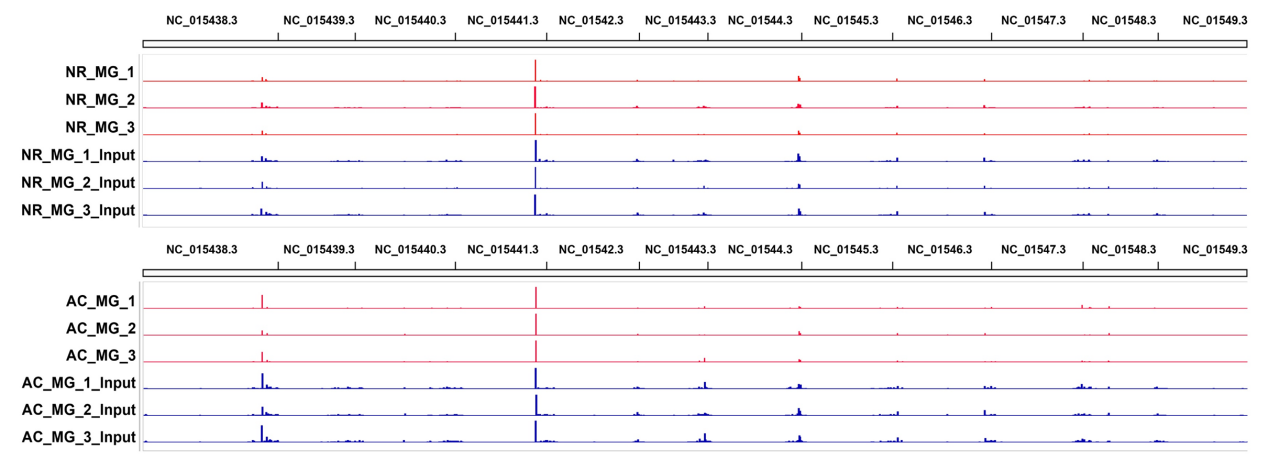


**Figure S8.** Integrated Genome Viewer (IGV) snapshots showing differences in mRNA acetylation (ac^4^C) between fruits from both genotypes at the MG stage.
